# Bovine ncRNAs Are Abundant, Primarily Intergenic, Conserved and Associated with Regulatory Genes

**DOI:** 10.1371/journal.pone.0042638

**Published:** 2012-08-06

**Authors:** Zhipeng Qu, David L. Adelson

**Affiliations:** School of Molecular and Biomedical Science, The University of Adelaide, Adelaide, South Australia, Australia; National Institutes of Health, United States of America

## Abstract

It is apparent that non-coding transcripts are a common feature of higher organisms and encode uncharacterized layers of genetic regulation and information. We used public bovine EST data from many developmental stages and tissues, and developed a pipeline for the genome wide identification and annotation of non-coding RNAs (ncRNAs). We have predicted 23,060 bovine ncRNAs, 99% of which are un-annotated, based on known ncRNA databases. Intergenic transcripts accounted for the majority (57%) of the predicted ncRNAs and the occurrence of ncRNAs and genes were only moderately correlated (*r* = 0.55, p-value<2.2e-16). Many of these intergenic non-coding RNAs mapped close to the 3′ or 5′ end of thousands of genes and many of these were transcribed from the opposite strand with respect to the closest gene, particularly regulatory-related genes. Conservation analyses showed that these ncRNAs were evolutionarily conserved, and many intergenic ncRNAs proximate to genes contained sequence-specific motifs. Correlation analysis of expression between these intergenic ncRNAs and protein-coding genes using RNA-seq data from a variety of tissues showed significant correlations with many transcripts. These results support the hypothesis that ncRNAs are common, transcribed in a regulated fashion and have regulatory functions.

## Introduction

As a result of advances in DNA sequencing technologies, a number of mammalian genomes have been sequenced and assembled. The impetus for sequencing mammalian genomes is to use comparative genomics to identify important, evolutionarily conserved sequences, such as protein-coding genes. While protein-coding genes are considered the most important elements of the genome, they only account for a small fraction of the genome sequence or the mammalian transcriptome. This indicates that the complexity of the mammalian genome, especially the transcriptome, cannot be interpreted merely according to the central dogma of molecular biology “DNA-RNA-protein” [1], [2], [3], [4], [5]. In human, only about 1–2% of the entire genome is transcribed as protein-coding RNAs, while more than half (∼57%) of the human genome is transcribed as “non-protein-coding” RNAs (ncRNAs) [3]. Furthermore, studies from the FANTOM consortium have also confirmed that the majority of the mouse genome is transcribed, commonly from both strands. Most of these transcripts cannot be annotated as protein-coding RNAs [4]. These findings are evidence of a hidden, non-protein-coding transcriptome in mammals.

At present there is debate about the true nature of the non-protein-coding transcriptome. Some believe that most ncRNAs are “transcriptional noise” associated with protein coding genes and have no function [6]. But this may not be the whole story. Apart from well-studied small non-protein-coding RNAs, like miRNAs, siRNAs, snoRNAs and piRNAs, other classes of abundant functional ncRNAs have been demonstrated in recent studies. Guttman *et al.* identified over a thousand highly conserved large intergenic non-coding RNAs (lincRNAs) in the mouse by analysing chromatin signatures [7]. Subsequent experimental analysis confirmed that one of these lincRNAs serves as a repressor in p53-dependant transcriptional responses [8]. Recently, another class of long non-coding RNAs was discovered in the human. Some of these thousand or so long ncRNAs were shown to have an un-anticipated enhancer-like role in activation of critical regulators of development and differentiation [9]. Furthermore, new types of small ncRNAs, like tiRNAs (tiny RNAs) [10], PASRs (Promoter-Associated Short RNAs) [11], TASRs (Termini-Associated Short RNAs) [11], and aTASRs (antisense Termini-Associated Short RNAs) [12], have been discovered in mammals. It is now clear that evidence confirms that there are indeed many functional sequences in the non-protein-coding transcriptome.

To characterize the non-coding transcriptome at genome scale, we built a computational pipeline to identify non-protein-coding transcripts from Expressed Sequence Tags (ESTs), which were originally designed to identify and annotate protein-coding genes. ESTs have the advantage of being readily available from public repositories, and are generally far longer than the RNA-seq tags generated by current high throughput DNA sequencers. The latter allows confident reconstruction of much longer transcripts. We used the bovine genome as a starting point for three main reasons: it has a large number of ESTs sampled from many tissues and developmental stages, the protein coding gene annotations are robust and based on thorough comparative genomic analysis and we had already exhaustively annotated the repetitive component of the genome [13]. We were thus able to reconstruct many long transcripts and unambiguously map them to either protein-coding genes or non-repetitive, non-protein-coding regions of the genome. In this report we have identified thousands of non-coding RNAs (ncRNAs), the vast majority of which were previously un-annotated. We have also characterized the genomic distribution of these ncRNAs, compared to protein-coding genes and carried out conservation analyses to detect evidence of potential conserved function. Our analyses show that most ncRNAs were transcribed from clearly conserved genomic regions. A predominant class of intergenic ncRNAs were transcribed from the proximate flanking regions of genes, leading us to hypothesize that they play *cis*-regulatory roles in the regulation of their neighbour genes and/or *trans*-regulatory roles elsewhere in the genome. Taken together, our findings provide a general view of the composition, distribution, and conservation of a mammalian non-protein-coding transcriptome at genomic scale, sampled across a wide selection of tissues and developmental stages, and support the idea that most ncRNAs are of potential functional importance.

## Materials and Methods

### Databases

All data used in this research were sourced from public databases. Bovine ESTs were retrieved from dbEST of NCBI [14]. The information from source libraries is shown in Table S1. Two different bovine repeat databases were used: the first was developed by Adelson *et al.* [13]; the other was a custom-built repetitive protein database generated according to Smith *et al.*'s method [15]. The genome assembly of bosTau4 and its corresponding RefSeq dataset (as of September of 2009) was downloaded from NCBI. The Swiss-Prot protein reference database (as of September of 2009) was also obtained from NCBI.

Several known ncRNA databases were used to annotate ncRNAs. The miRNA database, miRBase release 14, which included 10,566 mature miRNAs and 10,867 pre-miRNAs, was obtained from miRBase (http://www.mirbase.org/) [16]. Rfam9.1, which contained tRNAs, rRNAs, snoRNAs, miRNAs, and other ncRNA models, was obtained from http://rfam.janelia.org/ [17]. NONCODE2.0 was obtained from http://www.noncode.org/ [18].

### Programs used to develop the pipeline of ncRNA identification

All programs used in the pipeline of ncRNA identification can be freely accessed from the Internet (Table S2). All of them are stand-alone versions running under the Linux environment. Perl was used to link them into a pipeline. All Perl scripts are available upon request.

### Annotation of ncRNAs

Several methods were used to annotate bovine ncRNAs. Similarity search was used to identify miRNAs from bovine ncRNAs. Blastn of ncRNAs against both mature miRNA and pre-miRNA databases was used to find transcripts of significant similarity to known mature miRNAs (identity >95%, coverage = 100%) and primary miRNAs (identity >95%, coverage >95%). Two steps were used to validate tRNAs from bovine ncRNAs. tRNAscan_SE was used to generate a list of tRNA candidates [19]. Only the candidates subsequently validated by Rfam were classified as known tRNAs [17].

The Stand-alone Rfam search was performed by a Perl script Rfam_scan.pl provided with Rfam [17]. Additionally, BLASTN against NONCODE2.0 was used to identify long known ncRNAs and piRNAs [18].

### Distribution analysis of ncRNAs

All 23,060 ncRNAs and 24,373 RefSeqs were mapped to the bosTau4 assembly. The numbers of ncRNAs and RefSeqs in 1 MB non-overlapping bins were counted to determine the density distribution. The Spearman correlation coefficient between the densities of ncRNAs and RefSeqs per 1 MB bin across the whole genome was calculated using the R package (v2.12.0).

### Positional bias analysis of intergenic ncRNAs

For each ncRNA, the closest gene model, either upstream or downstream, was defined as the nearest neighbour. The intergenic region of two nearby genes was defined as the gene interval.

To maximize the number of intergenic ncRNAs annotated in this step, the transcription orientations of intergenic ncRNAs were determined by the union, instead of the intersection of the two methods used to determine the transcription orientation of ESTs in the step of *cis*-NATs (Natural Antisense Transcripts) identification.

### Functional over-representation of intergenic ncRNAs' neighbour genes

All neighbour genes with intergenic ncRNAs in 5 kb flanking upstream or downstream regions were identified. 3,166 unique genes with intergenic ncRNAs in 5′ flanking regions were identified, and 741 unique genes were identified with intergenic ncRNAs in 3′ flanking regions. The intersection of these two gene lists resulted in 183 unique genes. The GO (Gene Ontology) functional annotation and clustering were conducted using DAVID (Database for Annotation, Visualization and Integrated Discovery) [20], [21]. Over-represented GO terms were filtered to contain at least 5 genes and FDR (False Discovery Rate)<0.05. Ten control gene lists for 5′ and 3′ neighbour gene lists were generated respectively. For each control list for 5′ end intergenic ncRNA, 741 genes were randomly selected from all the genes with 5′ intergenic regions. For each control list for 3′ end intergenic ncRNA, 3,166 genes were randomly selected from all the genes with 3′ intergenic regions. All over-represented GO terms (≥5 genes and FDR<0.05) were highlighted as yellow in Table S3.

### Analysing the sequence conservation of predicted ncRNAs

Conservation analysis based on phastCons score [22]: The reference phastCons score files containing the phastCons scores for multiple alignments of 4 other vertebrate genomes (Dog, May 2005, canFam2; Human, Mar 2006, hg18; Mouse, July 2007, mm9; Platypus, Mar 2007, ornAna1) to the reference of cow genome (Oct 2007, bosTau4) were downloaded from UCSC (http://hgdownload.cse.ucsc.edu/goldenPath/bosTau4/phastCons5way/). Each base in the EST or RefSeq was assigned a phastCons score according to the reference files. The bases that were not included in the conserved elements of the reference files were given phastCons scores of “0”. For a given sequence, the mean phastCons score was calculated by normalizing the sum of phastCons scores against the length of the sequence.

Conservation analysis based on GERP++ score [23]: GERP++ is another tool that uses maximum likelihood evolutionary rate estimation for position-specific scoring. It calculates the RS (rejected substitution) score based on multiple alignments and a phylogenetic tree. The 5-way multiple alignment file for cow (the same species and genome assemblies used for phastCons scores) and the corresponding phylogenetic tree were downloaded from UCSC (http://hgdownload.cse.ucsc.edu/goldenPath/bosTau4/multiz5way/). A PERL script was created to convert the default multiple alignment file format into the file format that can be fed into GERP++. The GERP++ score for each base of bosTau4 was calculated using GERPv2.1 (http://mendel.stanford.edu/SidowLab/downloads/gerp/index.html). Mean GERP++ scores were calculated in the same way as mean phastCons scores.

24,000 genomic fragments, which ranged in size from 500 bp to 15,000 bp, were randomly extracted from un-transcribed regions of bosTau4 as the control dataset. The cumulative frequency for each dataset was calculated and plotted using the R package.

### Identification of sequence specific motifs from intergenic ncRNAs

Bovine gene expression profiles were generated based on transcriptome data from 95 samples (92 adult, juvenile and fetal cattle tissues and 3 cattle cell lines) [24].

FIRE was used to predict sequence motifs from bovine intergenic ncRNAs [25]. Bovine intergenic ncRNAs located in 5 kb of upstream or downstream gene flanking regions were used as motif prediction pools. Intergenic ncRNAs were converted as sense RNAs according to their transcription orientation. The motif-identification mode was set as “DNA”, which means motif sequence can be predicted from both strands of intergenic ncRNAs. FIRE was run against 5′ end and 3′ end intergenic ncRNAs according to 95 individual gene expression profiles respectively.

The comparison of predicted RNA sequence motifs against known DNA motifs was performed using the TOMTOM web server [26].

### Expression correlation analysis based on bovine MPSS data

The expression profiles of intergenic ncRNAs and bovine RefSeqs were calculated based on the MPSS (Massively Parallel Signature Sequencing) tags mapped to the 3′ most end of each transcript [24]. The tag count for each transcript was normalized according to the library size. Transcripts mapped with less than 3 tags were removed from the expression profile. The MIC score was generated by MINE based on the expression of intergenic ncRNA and RefSeq pairs [27]. Only intergenic ncRNAs/RefSeqs with expression (read counts) in at least 3 libraries were used to perform expression correlation analysis.

## Results

### The development of ncRNAs identification pipeline

**Figure 1:**
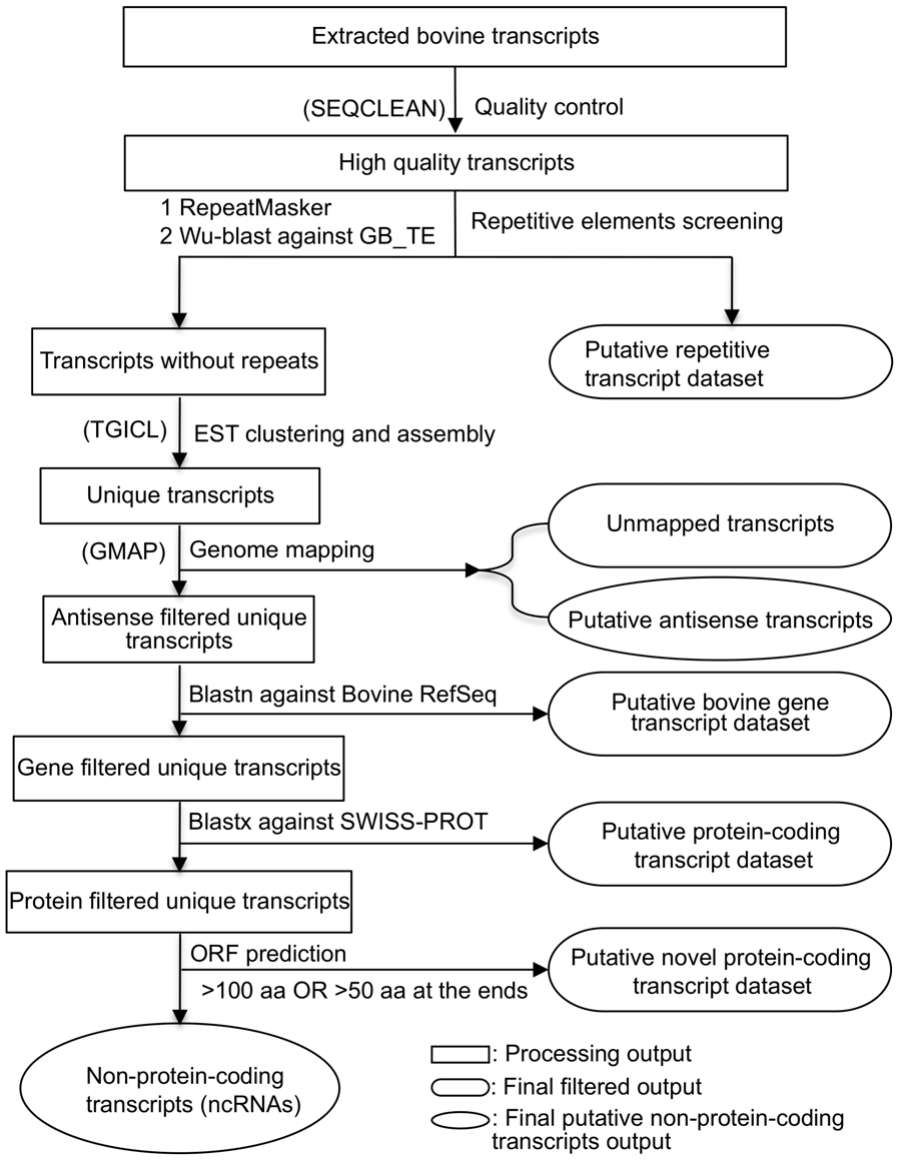
Flowchart describing the pipeline for ncRNA identification.

We identified ncRNAs from bovine ESTs, by developing a computational pipeline based on public software and Perl scripts (Figure 1). A total set of 1,517,143 bovine ESTs (as of 30^th^ September, 2009), extracted from the dbEST of NCBI, was processed as the input dataset for the pipeline. After quality control, repeat filtration and EST assembly, we identified 216,095 unique transcripts. We opted for stringent mapping criteria (coverage ≥90% and identity ≥95%) and as a result, 69,099 unique transcripts were unable to be mapped to the BosTau4 assembly and were therefore discarded. Of the mapped sequences, 3,121 were classified as putative *cis*-NATs, 74 of which were subsequently manually checked on UCSC genome browser (Materials S1). The remaining 143,875 mapped unique transcripts were further analysed to annotate and characterize the bovine transcriptome.

Of the 143,875 mapped unique transcripts, 87,373 were very similar to bovine RefSeqs (E-value<1e-3), and 48,773 of them shared similarity over more than 90% of their length with 14,962 RefSeqs and were denoted as known gene transcripts. Of the 38,600 sequences that shared similarity with RefSeqs over less than 90% of their length, more than one third (13,035) were un-spliced.

There were 1,856 transcripts, which we were unable to annotate based on similarity search against bovine RefSeqs, but were identified by BLAST in the Swiss-Prot database at the amino acid level. These sequences may represent novel un-annotated bovine protein-coding genes that are conserved across taxa.

The resulting set of sequences, filtered with respect to sequence similarity to repeats, protein-coding transcripts and *cis*-NATs was then further scrutinized by checking the length of predicted ORFs (Open Reading Frames). As a result, 31,586 unique sequences were removed from the 54,646 “protein-coding gene filtered unique transcripts” because they contained either long predicted ORFs (≥100 amino acids) or shorter ORFs (≥50 amino acids) at the ends. These “ORF-containing sequences” may include transcripts from un-annotated, novel protein-coding genes. The large number of these transcripts raises the possibility that there are still significant numbers of protein-coding genes in the bovine genome that remain undiscovered.

As a result of this highly stringent filtering against known protein-coding genes and the exclusion of ORF containing transcripts we were left with 23,060 ncRNAs (Table S4), which accounted for ∼15.5% (23,060 out of 143,875) of the mapped bovine unique transcripts. These ncRNAs were then analysed to identify previously annotated ncRNAs.

### Few well-characterized ncRNAs were identified

The annotation of the 23,060 ncRNAs was carried out using several different methods (See methods for detailed procedures). As a result of this effort we determined that only 77 of these sequences had been previously identified as ncRNAs, either as miRNAs, snoRNAs, tRNAs, rRNAs, mRNA-like ncRNAs, piRNAs and other ncRNAs (Materials S1, Table S5 and Table S6). One additional class of ncRNAs that we identified were *cis*-NATs. We identified 74 *cis*-NAT*s* distributed on 28 different chromosomes (Materials S1 and Table S7 and Figure S1).

Whilst our results showed that ESTs could be used to identify ncRNAs by rational and stringent sequence similarity searches, the vast majority of the ncRNAs we identified could not be annotated based on previously well-characterized ncRNAs.

### Genome-wide distribution of ncRNAs

**Figure 2:**
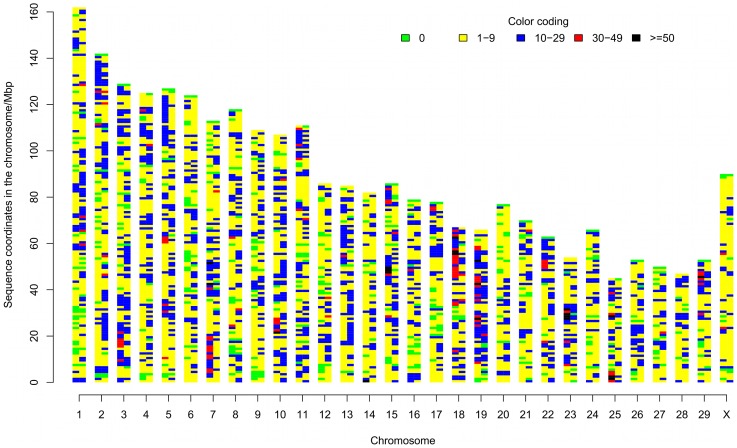
Distribution of genes and ncRNAs in the bovine genome. Chromosomes are on the X axis, and sequence coordinates on the Y axis, with the “top” of the chromosome at the Y axis origin. All cattle autosomes are acrocentric. Each chromosome is represented by two vertical bands, the left band shows gene number and the right band shows ncRNA number, per 1 Mb bin. The legend shows the band colour coding for numbers per 1 Mb bin.

**Figure 3:**
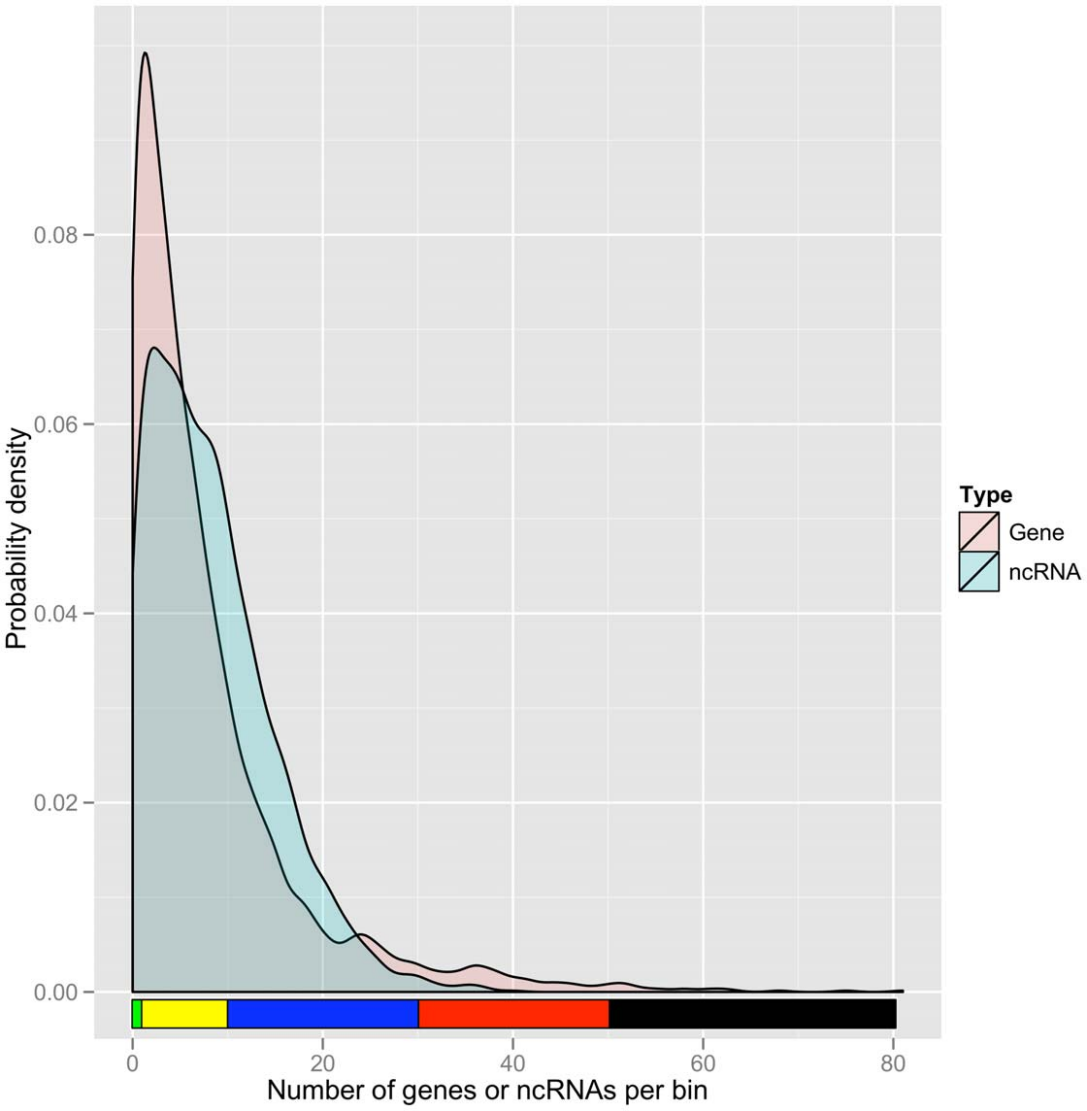
Probability densities of genes and ncRNAs per 1 Mb bin. NcRNAs have similar genomic densities compared to protein coding genes, but with fewer extreme density regions. The colour coding is consistent with Figure 2.

To understand the distribution of predicted ncRNAs in the genome, our 23,060 predicted ncRNAs mapped onto BosTau4 were compared to the mapped locations of 24,373 bovine RefSeqs. Figure 2 shows the density distributions of ncRNAs and RefSeqs in 30 bovine chromosomes (29 autosomes and X). Together with the relative frequencies of the densities of ncRNAs and RefSeqs, which are shown in Figure 3, it is obvious that the “gene poor regions” (with fewer than 10 genes in 1 Mb) are more abundant than “ncRNA poor regions” (less than 10 ncRNA s in 1 Mb) in the bovine genome. Furthermore, 288 gene deserts (no gene in 1 Mb) were identified compared to 156 ncRNA deserts (no ncRNA in 1 Mb). At the other end of the gene density spectrum, 21 regions were found with more than 50 genes/Mb, but no comparable regions were found for ncRNAs. These results showed that ncRNAs were more evenly distributed than protein-coding genes across the genome. A correlation analysis of the densities of protein-coding genes and ncRNAs per 1 Mb revealed only a moderate correlation between these two transcriptome sets at the whole genome level (*r* = 0.5528816, *p*<2.2e-16).

**Figure 4:**
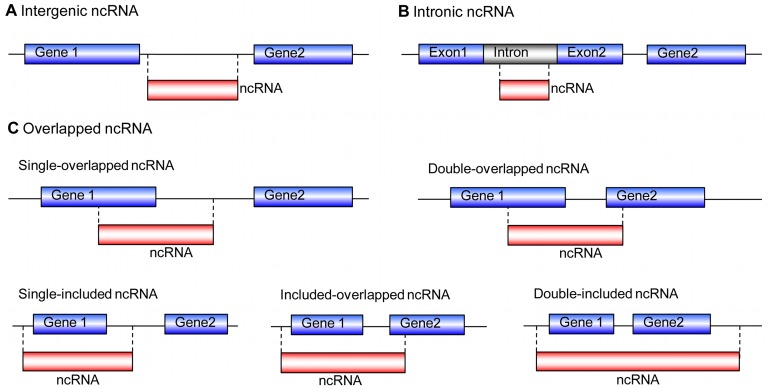
Classification of ncRNAs in relation to protein-coding genes. (A) The entire EST is transcribed from an intergenic region, regardless of the transcription orientation. (B) The entire EST is transcribed from an intron, regardless of the transcription orientation. (C) Single-overlapped ncRNA: EST partially overlapped with a gene; Double-overlapped ncRNA: Both ends of the EST overlapped with two genes and spanned an intergenic region; Single-included ncRNA: The gene was fully included inside the EST; Included-overlapped ncRNA: One gene was fully included within the ncRNA, and the ncRNA spanned the intergenic region and overlapped with a neighbour gene; Double-included ncRNA: More than one genes were fully included within the EST.

**Figure 5:**
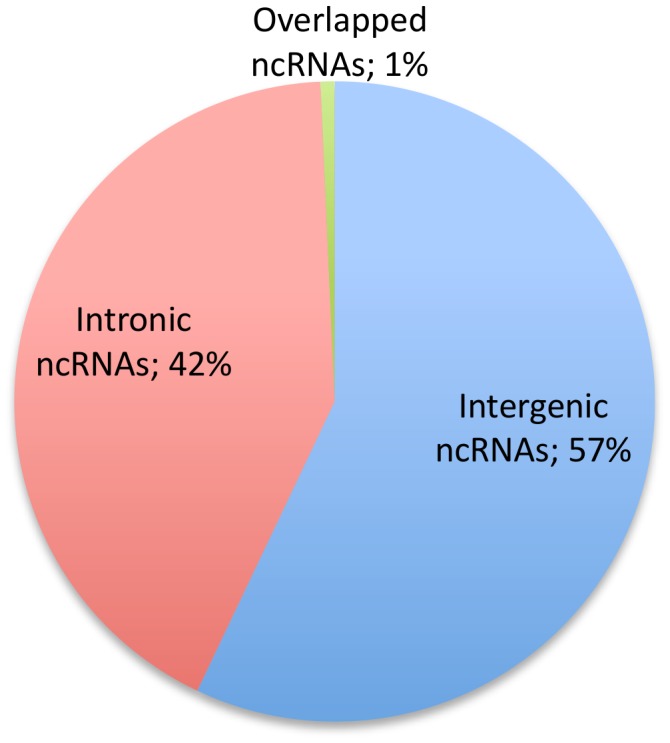
Relative abundance of the three main classifications of ncRNAs. Almost 60% of ncRNAs are long intergenic non-coding RNAs (intergenic ncRNAs).

We further classified our ncRNAs with respect to neighbour protein-coding genes to analyse the potential transcriptional overlap with RefSeq genes. Our classification scheme for ncRNAs is shown in Figure 4. Excluding 952 ncRNAs mapped to uncharacterized genomic locations, there were three main types of ncRNAs based on this classification and their relative proportions are shown in Figure 5. The majority of the ncRNAs in our dataset were intergenic transcripts (57% intergenic compared to 42% intronic). We also noticed that most ncRNAs were singletons (72.2% out of intergenic, 81.1% out of intronic and 71.3% out of overlapped ncRNAs respectively)(Table 1). The data in Table 1 also showed that the vast majority of ncRNAs (both intergenic and intronic) were apparently unspliced transcripts.

**Table 1:** Summary of transcriptional redundancy and splicing information of three types of ncRNAs.

Class of ncRNAs	Number	Singleton		Unspliced	
		Count	Fraction	Count	Fraction
Intergenic	12,614	9,113	72.2%	9,852	78.1%
Intronic	9,337	7,571	81.1%	8,085	86.6%
Overlapped	157	112	71.3%	80	51.0%
–Single-overlapped	138	96	69.6%	78	56.5%
–Double-overlapped	2	2	100%	0	0
–Single-included	10	9	90%	1	10%
–Included-overlapped	2	2	100%	0	0
–Double-included	5	3	60%	1	20%

– denotes subclass of Overlapped.

Detailed inspection of overlapped ncRNAs revealed that 98 of them overlapped with their corresponding genes by less than 50 basepairs; 85 of them at the 3′ end, and the rest at the 5′ end of the genes. These ncRNAs may represent unannotated UTRs or 5′ and 3′ extensions of genes [28], but there is the possibility that some of them, especially 5′ overlapped ncRNAs, were transcribed as functional ncRNAs, like PASRs, tiRNAs or uaRNAs [10], [11], [29], [30]. Our result did show that there are antisense transcripts among these overlapped ncRNAs (10 of 85 at 3′ end and 3 of 13 at 5′ end).

### Most ncRNAs were of intergenic origin

**Figure 6:**
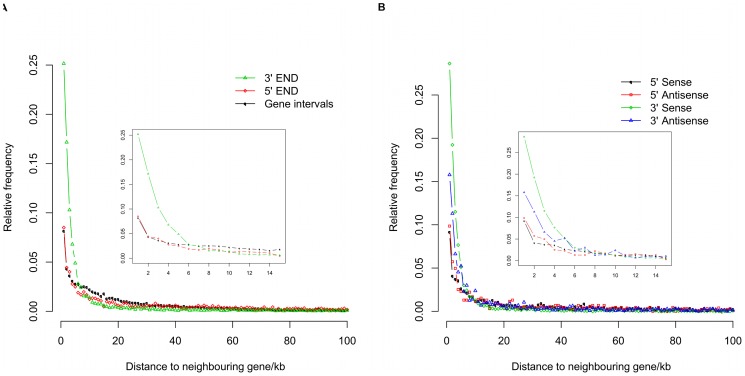
Positional bias distribution of ncRNAs with respect to neighbour genes. (A) Relative frequencies of ncRNAs with respect to the distance from neighbour genes. 100 kb adjacent to TSS or TTS of genes is shown in these plots. 3′ END means the ncRNA is located in the 3′ flanking region of its neighbour gene. 5′ END means the ncRNA is located in the 5′ flanking region of its neighbour gene. “Gene intervals” refers to the intergenic region of two adjacent genes. (B) Relative frequencies of ncRNAs from neighbour genes partitioned with respect to transcription orientation. The internal boxes represent the zoom in view of the relative frequencies from 5 kb to 20 kb.

Most bovine ncRNAs mapped to intergenic regions (Figure 5). To get a better understanding of these intergenic ncRNAs, we plotted the frequency distribution of intergenic ncRNAs as a function of their distance and transcriptional orientation to the nearest neighbour genes (Figure 6). About 67.4% (8,500 out of 12,614) of intergenic ncRNAs had a neighbour gene within 20 kb, with a significant concentration of intergenic ncRNAs in the 5 kb flanking regions of genes. Beyond 10 kb, the number of intergenic ncRNAs decreased very gradually as a function of distance. It was also apparent from Figure 6A that intergenic ncRNAs were more prevalent at the 3′ end of genes than at the 5′ end. The intergenic ncRNAs closest to the 5′ end of a gene also tended to be within 5 kb of the gene, but this localization was not significantly different to the control frequency distribution calculated using gene to gene nearest neighbour distances, where the majority of intergenic distances were less than 5 kb. We were able to determine transcriptional orientation of 10,969 of 12,614 intergenic ncRNAs based on their dbEST annotation. When we compared the transcriptional orientation of these intergenic ncRNAs to their closest gene neighbour, we observed that most of them closest to the 3′ end of genes were transcribed from the same strand as the gene (Figure 6B). There were four times more ncRNAs in the same transcriptional orientation when they were 3′ to the closest gene (6,296 to 1,433). This difference in transcriptional orientation for the ncRNAs 5′ of the closest gene was also observed, but not to the same degree (1,931 same to 1,309 reverse). The intergenic ncRNAs, transcribed from the same strand as the closest gene, might be extensions of the UTRs produced by alternative transcription start or termination sites of protein-coding genes, but many of them were at significant distances from these genes making this an unlikely possibility.

To determine the likelihood that these intergenic ncRNAs were potential gene UTRs, we compared them against the annotated UTR database (including human, mammals and vertebrates) [31]. 3,168 of these intergenic ncRNAs were highly similar to 3′ UTRs (E-value<1e-3), while only 198 were highly similar to 5′ UTRs (E-value<1e-3). Together with 2,516 intergenic ncRNAs which are located in the proximal 1 kb of gene flanking regions (5′ end or 3′ end), we classified these 4,584 intergenic ncRNAs as UTR-Related RNAs (Table S4), which are named to differentiate them from uaRNAs (UTR-associated RNAs), a class of previously annotated independent ncRNAs transcribed from UTRs [30]. The reasonably large number of intergenic ncRNAs transcribed in the opposite orientation to their nearest gene (1,309 from the 5′ end and 1,433 from the 3′ end), raised the possibility that there might be transcriptional antisense regulation associated with these elements.

The spatial clustering of all predicted intergenic ncRNAs with respect to protein coding genes suggested a *cis*-regulatory relationship to us. To understand the potential biological significance of such a relationship, we functionally clustered the neighbour genes within 5 kb flanking regions of intergenic ncRNAs according to GO [32]. We found that regulatory genes were over-represented in the neighbour genes of these intergenic ncRNAs (Table S3), but the gene count of these over-represented GO terms was very small, most likely because of the poor functional annotation of bovine reference genes in GO. The functional clustering of control gene lists (see methods) indicated these over-representations were not chance occurrences (Table S3). When we differentiated the neighbour genes according to the position of their nearby intergenic ncRNAs, we observed that positive regulatory genes were over-represented in the neighbour genes with intergenic ncRNAs in their 5′ flanking regions (Table S3). Assessment of neighbour gene function based on regulatory-related keywords searching of the subset of 183 genes flanked at both ends by intergenic ncRNAs revealed that 85 (46.4%) of these genes were involved in either transcriptional regulation, signal transduction or encoded domains consistent with these functions. By comparison, only 8,087 (33.2%) of all 24,373 RefSeq genes were annotated as regulatory genes based on the same keywords searching. This indicated that the purely GO-based results were probably a significant underestimate of the regulatory potential of these neighbour genes. In summary, we hypothesize that our gene-proximate intergenic ncRNAs are potentially *cis*-regulatory and tend to regulate regulatory genes. Confirmation of this hypothesis will have to await specific, functional perturbation experiments, but is consistent with published data from small numbers of intergenic ncRNAs.

### Evolutionary conservation of bovine ncRNAs

**Figure 7:**
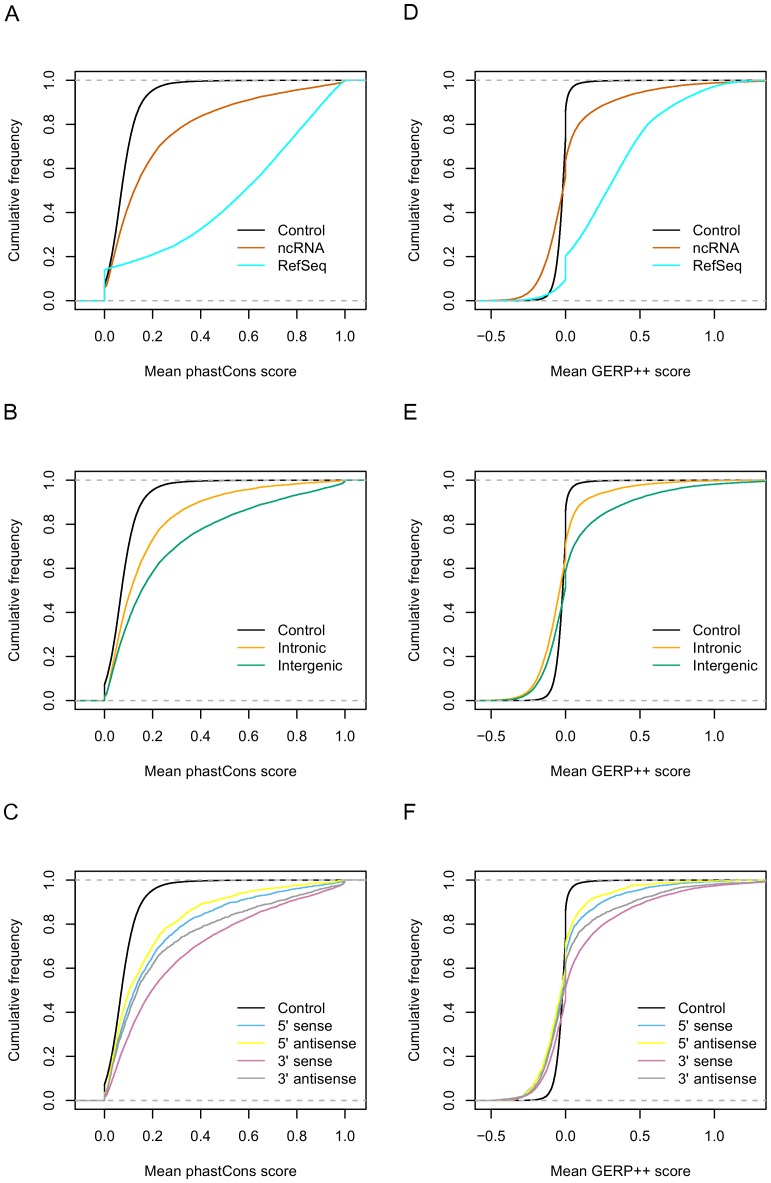
Sequence conservation analysis of ncRNAs. (A, B & C) are based on phastCons score. (D, E & F) are based on GERP++ score. The control line is based on a similar number of randomly selected non-transcribed genomic regions. A & D – ncRNAs compared to RefSeqs, B & E – intergenic ncRNAs compared to intronic and C& F – 5′ vs 3′ ncRNAs and transcriptional orientation with respect to nearest neighbour genes.

To assess whether ncRNAs were under selective constraint, we used two different methods to assess the degree of sequence conservation as shown in Figure 7. Figure 7A shows the degree of conservation based on phastCons score; ncRNAs were clearly conserved compared to control sequences, which were selected at random from un-transcribed regions of the bovine genome, but were less conserved compared to protein-coding genes. When we compared the degree of sequence conservation between intergenic and intronic ncRNAs according to phastCons score (Figure 7B), intergenic ncRNAs were more conserved than intronic ones. When we further refined this to assess the sequence conservation of intergenic ncRNAs according to their relationships with protein-coding genes, we observed that intergenic ncRNAs closest to the 3′ end of genes were more conserved than those closest to the 5′ end of genes. And when we took into the consideration the transcriptional orientation of these ncRNAs with respect to their closest gene, the “sense” intergenic ncRNAs, which are transcribed from the same strand as their neighbour genes, were more conserved than the “antisense” intergenic ncRNAs, regardless of whether they were closest to the 5′ or 3′ end of protein-coding genes (Figure 7C).

We were able to confirm these observations regarding the conservation level of ncRNAs using GERP++ [23], based on a different statistical model. If we only consider the sequences that were under a substitution deficit (positive score), the conservation level of ncRNAs was between protein-coding genes and un-transcribed genomic fragments, which was consistent with the phastCons result. Nearly 40% of ncRNAs had a substitution deficit, compared to ∼80% of protein-coding genes and less than 20% of un-transcribed genomic fragments. On the other hand, for sequences that showed a substitution surplus (negative score), the divergence level of ncRNAs was more pronounced than for protein-coding genes and un-transcribed genomic fragments (Figure 7D). The results of the GERP++ score for the intergenic and intronic ncRNAs, as well as the different intergenic classes were also consistent with their respective phastCons score results (Figure 7E and Figure 7F).

When we removed all UTR-related RNAs from 23,060 ncRNAs, the remaining sequences still showed clear conservation compared to un-transcribed control fragments (Figure S2). The highly conserved UTR-related RNAs is consistent with these being part of poorly annotated UTRs or independent transcripts from UTRs, as UTRs across different species are often well conserved (Figure S2).

### Identification of sequence motifs from intergenic ncRNAs

**Figure 8:**
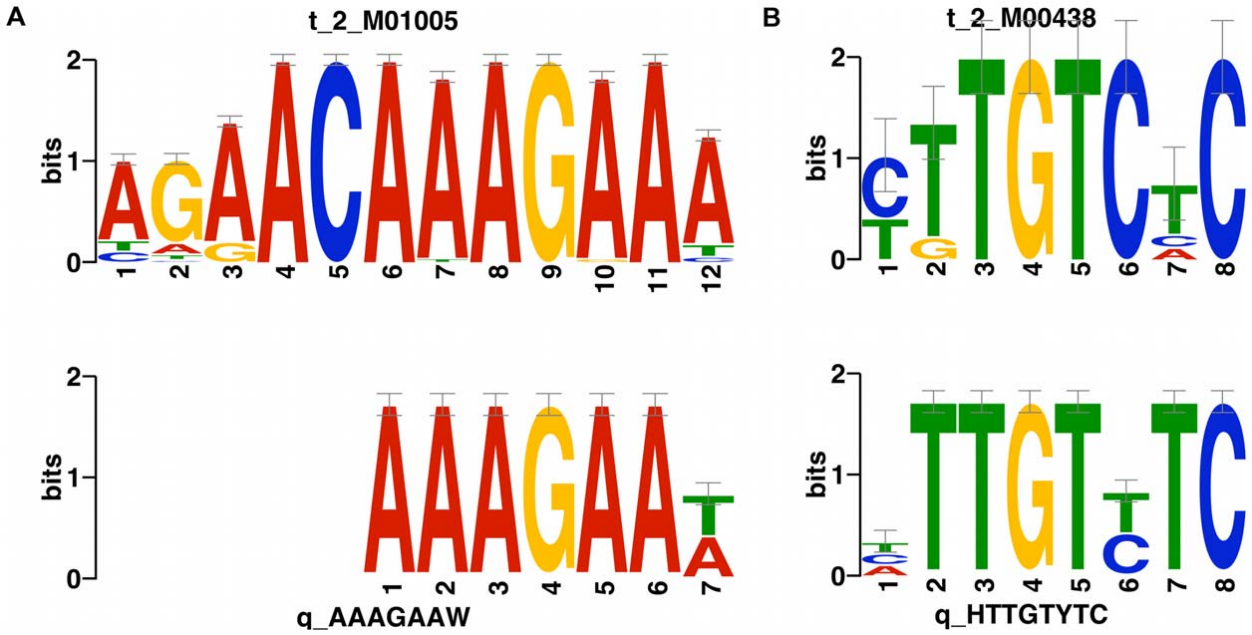
Two sequence motifs from intergenic ncRNAs with significant similarity against known DNA motifs. For each comparison, the upper one is the known DNA motif, and the lower one is the intergenic ncRNA sequence motif.

Based on the gene expression profiles generated from 95 bovine transcriptome libraries, we identified 21 sequence specific motifs from 5′ intergenic ncRNAs and 29 from 3′ intergenic ncRNAs (Table S8, A & B). By comparison against known DNA motif databases using TOMTOM, we found that 2 motifs, “160_1_5END” from 5′ end intergenic ncRNAs and “086_1_3END” from 3′ end intergenic ncRNAs, showed significant similarity against known DNA motifs “ste11” and “ARF” respectively (p-value<1e-04 and FDR<0.05) (Figure 8 and Table S8). It is interesting to note that the number of “sense” sequence motifs of “ste11” (the motif is the same as the intergenic ncRNA strand) is almost equal to the number of “antisense” “ste11” motifs (the motif is complementary to the intergenic ncRNA strand) (Table S8, A & B). 3 other motifs from 5′ intergenic ncRNAs and 4 from 3′ intergenic ncRNAs also showed strong similarity (p-value<1e-04, FDR<0.5) against known DNA motifs (Figure S3 and Figure S4). The numbers of “sense” and “antisense” sequence sites in intergenic ncRNAs are almost equal for most of the identified motifs (Table S8, A & B and Figure S5).

After we removed all UTR-related RNAs from the 5 kb intergenic ncRNAs and re-ran the motif identification procedure with the same expression profiles and parameters, we still found 15 and 17 motifs from the remaining 5′ and 3′ intergenic ncRNAs. However, all of these novel 32 motifs were different to the 50 originally identified motifs (Table S8, C & D). Only one novel 3′ motif (136-1, [ACT]AG[AC]CATA[AGT]) showed similarity with a known DNA motif FOXL1, which was also the best hit for an originally identified 3′ end motif (119_1_3END, [ACT]AAA[CT]ATA[GT]).

### Expression correlation and functional significance

**Figure 9:**
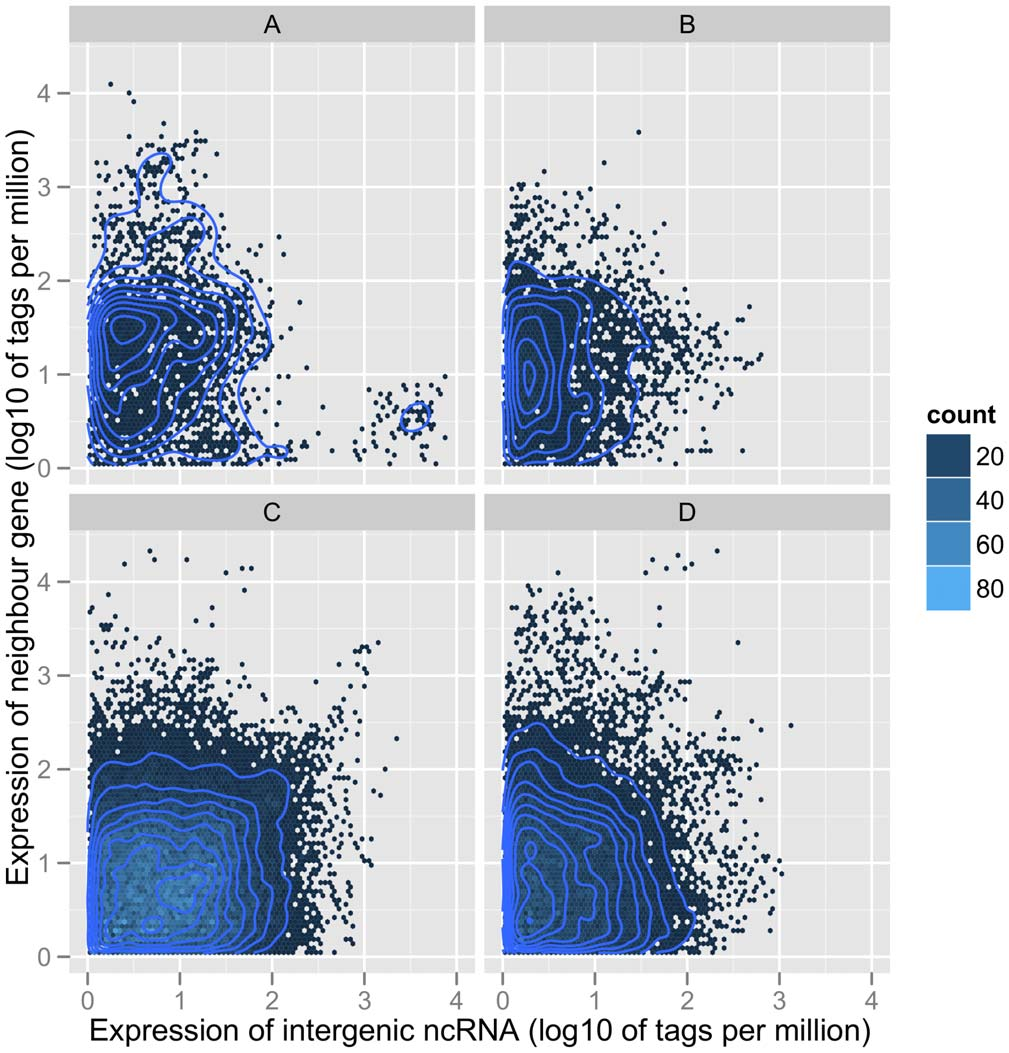
Scatter plot for the log10 ratio of expressions of intergenic ncRNAs and corresponding neighbour genes. Dots were binned into 80*80 hexagons across the plot area. Different colours represent the dot count in each bin. A represents the expression of 5′ end UTR-related RNAs and neighbour genes. B represents the expression of 5′ end intergenic ncRNAs with UTR-related RNAs removed and neighbour genes. C represents the expression of 3′ end UTR-related RNAs and neighbour genes, and D represent the expression of 3′ end intergenic ncRNAs with UTR-related RNAs removed and corresponding neighbour genes.

Most of the identified intergenic ncRNAs reported from other species were directly or indirectly involved in gene regulatory networks. To understand whether there are correlations between the expression of intergenic ncRNAs and corresponding neighbour genes, we identified all intergenic ncRNA and neighbour gene pairs with expression in at least one library based on the 95 bovine MPSS transcriptome data. Globally, there was no clear correlation between the expression of intergenic ncRNAs and corresponding neighbour genes no matter whether intergenic ncRNAs were at the 5′ end or 3′ end of the genes (Figure 9). Because many intergenic ncRNAs containing sequence motifs are also close to regulatory genes, we checked the expression of these “motif and regulatory” intergenic ncRNAs across different libraries (Figure S6). Some of these intergenic ncRNAs showed negative expression correlation with neighbour genes. One of these intergenic ncRNAs is the antisense transcript of protein-coding gene “*ZNFX1*” (Figure S6). In human, the antisense transcript of “*ZNFX1*” has been annotated as “*ZNFX1-AS1*” [33]. This antisense transcript in bovine might be the homolog of the human “*ZNFX1-AS1*”. This bovine *“ZNFX1-AS1”* does not show high sequence conservation with 4 different human transcript variants (Figure S7). It is also the host transcript of two possible snoRNAs (SNORD12 and SNORD12B), which is consistent with human *“ZNFX1-AS1”* (Figure S8) [33].

To understand the associations between the expression of intergenic ncRNAs with other protein-coding genes, we used MINE (Maximal Information-based Nonparametric Exploration) to analyse the correlations between each intergenic ncRNA and all RefSeq genes [27]. For most intergenic ncRNAs detected by the RNA-seq data (191 out of 389 at 5′ end and 1,678 out of 2,673 at 3′ end), we identified significantly associated protein-coding genes based on MIC (Maximal Information Coefficient) score, with FDR≤0.05 after multiple testing (Table S9), and many of these showed significant associations with multiple protein-coding genes in terms of their expression, with 35 out of 191 5′ intergenic ncRNAs and 425 of 1,678 3′ end intergenic ncRNAs correlated with their neighbour genes (Table S9). 78 of the 191 5′ intergenic ncRNAs and 1,124 of the 1,678 3′ end intergenic ncRNAs were UTR-related RNAs.

## Discussion

### Identification of ncRNAs

While increasing numbers of studies have confirmed that ncRNAs possess significant regulatory functions in different biological pathways, their computational identification can be very challenging. One current approach is to identify ncRNA based on homology searches, such as sequence-based, profile HMM and structure enhanced methods [34], [35], [36]. Compared to these methods, our pipeline for ncRNA identification has two advantages [37]. First, our ncRNAs were identified from transcriptome data. Most homology-search-based methods use the entire genome sequence as the starting point, so it is not obvious if the ncRNAs identified by these methods are transcribed functional elements. Normally, further experiments are required to validate the expression of these functional elements. Second, most of the homology search methods are based on multi-alignments or taking known ncRNAs as a training set, so the output generated by these programs tends to identify only conserved ncRNAs. Conservation of ncRNAs is not as obvious as mRNAs. Some ncRNAs, like miRNAs, are indeed under strong selective constraint, but more ncRNAs, especially long ncRNAs, seem to be less conserved than protein-coding RNAs. By using stringent filters in our pipeline, we effectively removed the protein-coding transcripts, and identified different kinds of ncRNAs, which were not restricted to conserved ncRNAs. For the time being we have ignored ncRNA transcribed from repetitive elements, mostly retrotransposons, because it is virtually impossible to map such sequences to a unique genomic location and conservation scores for such sequences are only available for ancestral retrotransposon insertions. However retrotransposon ncRNAs may also be functional, as previous investigators have shown that transcripts of retrotransposon origin are differentially regulated during development [38].

The existence of well-characterized ncRNAs in our ncRNA dataset indicated that our pipeline was effective but also illustrated how few ncRNAs were conserved on the basis of sequence similarity. To avoid false positives, we relied on stringent criteria. For example, when mapping transcripts to the genome, only transcripts mapped with more than 90% coverage and greater than 95% identity were kept for further analyses. This explains why approximately 32% of the unique transcripts were classified as “un-mapped” transcripts. These criteria ensured that we removed contaminating and error rich sequences. Subsequently, when filtering protein-coding genes using BLAST, transcripts with hits (E-value<1e-5), regardless of coverage or percent identity in bovine RefSeq or Swiss-Prot databases, were discarded. This ensured that un-annotated distant paralogs or pseudogenes along with protein-coding ESTs were removed from our ncRNA set.

As a result, our pipeline provides a tool to mine the abundance of ESTs, which were originally used to identify protein-coding genes. Many studies have confirmed that ESTs can be used to detect ncRNAs. The most important evidence is the FANTOM ncRNA dataset, which are mRNA-like ncRNAs identified from mouse cDNAs [4]. NcRNAs identified from ESTs have also been reported in other organisms [39], [40]. Recently, a class of human long ncRNAs with enhancer-like function was identified from GENCODE annotation that, in part, relied on ESTs mapped to non-protein-coding regions [9]. Because our analyses were based on such stringent criteria, it is quite likely that our results represent a conservatively low estimate of the number of long ncRNAs in a mammalian transcriptome.

### The genome-wide distribution of ncRNAs

According to previous RNA-seq and tiling-array studies, more reads can be mapped to intronic than intergenic regions [5]. In contrast, our data showed that there were more intergenic than intronic ncRNAs in the bovine non-protein-coding transcriptome. Introns are known to be rich sources of both small and long ncRNA transcripts [41], but the larger number of conserved intergenic ncRNAs that we identified indicated that there might be more functional regulatory transcripts embedded in the intergenic regions of bovine genomes.

Previous research has shown that many ncRNAs are expressed in tissue-specific fashion or are restricted to certain developmental stages [42], [43], [44], which would likely manifest as singletons in the pooled tissue, normalized EST libraries that account for almost all of the bovine ESTs we analysed. Furthermore, the prevalence of unspliced transcripts (Table 1) was also reported in ncRNAs by Khachane *et al.* in a dataset of functional long ncRNAs [45]. These features may explain that why ncRNAs are not as easily detected as protein-coding genes in many situations.

The genome-wide map of ncRNA distribution in bovine demonstrates that ncRNAs are more evenly spread throughout the genome than protein-coding genes. This may mean that ncRNAs have evolved differently to protein-coding genes, which can form gene-rich regions by gene duplication [46]. This might also partially explain the poor conservation of ncRNAs. The different genomic distributions of ncRNA compared to genes is reflected in the moderate correlation between the densities of ncRNAs and protein-coding genes, indicating that many ncRNAs may function as remote regulatory elements rather than regulating their neighbour genes in some proximity based fashion. Previously, ncRNAs have been experimentally demonstrated to regulate gene expression by influencing the transcription process or chromatin structure in *trans*-acting fashion [47], [48], [49]. Some of these newly discovered enhancer-like long ncRNAs activate distant genes rather than surrounding ones, at distances in excess of 300 kb [9].

The moderate correlation of ncRNA density with gene density is also reflected in the fact that most bovine intergenic ncRNAs were transcribed from regions near protein-coding genes, especially from the 3′ end. This distribution bias has been observed previously in RNA-seq and tiling array expression experiments [4], [29], [50]. Our results however, were based on long reads from most tissues and developmental stages and were therefore unlikely to result from short, ragged ends of run-on transcripts. Furthermore, while many of these transcripts were found very near to genes, significant numbers were also found thousands to tens of thousands of base pairs away. Even in the UTR-related RNAs that we classified, there are still a proportion (492 of 4,584) transcribed from the antisense strand of protein-coding genes. Therefore, most of the intergenic ncRNAs, which were transcribed from both strands near protein-coding genes were inconsistent with trivial explanations such as transcriptional noise or mis-annotated UTRs. We therefore need to consider that these gene proximate intergenic ncRNAs may function as either *cis*-regulatory elements of their neighbour genes or as *trans*-acting regulatory sequences. Previous studies have confirmed that there are functional ncRNAs transcribed from the promoter, transcription start and terminal regions of protein-coding genes in sense orientation [10], [11]. Evidence for antisense ncRNAs comes from a recent study, using tSMS (true Single Molecule Sequencing) technology [12], [29]. In this study, a novel RNA copying mechanism was proposed, capable of producing antisense poly(U) small RNAs from the transcription start or terminal regions of genes, confirming that some human ESTs result from this process [12]. This is consistent with our results, where a significant fraction of the gene-proximate antisense ncRNAs were mapped very close to the 3′ ends of genes. However, while the functional significance of such antisense transcripts is unknown, this copying mechanism does not explain the significant fraction of gene proximate ncRNAs originating from the antisense strand much further away from the 3′ ends of genes. Even for the intergenic ncRNAs close to 3′ end neighbour protein-coding genes, in the same transcriptional orientation, which might be transcribed from potential un-characterized UTRs, there is also the possibility that they are independent functional transcripts, which have been observed mostly in human, mouse and fly genomes, and classified as uaRNAs [30]. On balance it is difficult to come up with a reasonable, consistent and trivial explanation for the occurrence of non-coding transcripts such as our ncRNAs leading us to conclude that they have a biological purpose.

### Conservation level of ncRNAs

The vast majority of the ncRNAs we have identified did not have detectable sequence similarity with well-annotated ncRNAs. However, in general, the conservation analysis of bovine ncRNAs based on phastCons and GERP++ score showed that ncRNAs were less conserved than protein-coding genes, while still exhibiting strong selection signatures. Our result was consistent with previous studies, which demonstrated that ncRNAs might experience different selective constraints compared to protein-coding genes [7], [9], [51]. Our result was also consistent with the possibility that ncRNAs might represent different ncRNA categories, each manifesting different levels of sequence conservation.

We observed that intergenic ncRNAs were slightly more conserved than intronic ones. This finding indicated that there might be more functional elements transcribed from the intergenic regions of the genome, such as recently discovered novel ncRNAs, including uaRNAs, PASRs, lincRNAs and enhancer-like RNAs, identified from intergenic regions [7], [9], [10], [11], [30].

### Sequence specific motifs identified from intergenic ncRNAs

Previous studies have reported that there are small or long ncRNAs transcribed from gene regulatory elements, like promoter regions. A report from Hans *et al.* showed that there are ncRNAs transcribed from promoter regions, which were named promoter-associated RNAs [52]. These promoter-associated RNAs function as recognition motifs to direct epigenetic silencing complexes to the promoter regions of target genes. Promoter-associated RNAs can also interact with transcription factor recognition sites to form DNA:RNA triplexes, which then interact with the rDNA promoter, mediating recruitment of DNMT3b and silencing rRNA genes by epigenetic regulation [53]. The location of these 5′ end bovine intergenic ncRNAs with respect to their corresponding neighbour genes and the existence of common sequence motifs indicate that these sequence motifs from intergenic ncRNAs may function as recognition sites for RNA-binding proteins, which form an RNA-protein complex to modulate target gene expression. Some sequence motifs from our 5′ end intergenic ncRNAs showed strong similarity with known DNA motifs and the almost equal numbers of sense and antisense motifs distributed in these transcribed 5′ end intergenic ncRNAs indicated that they might be compatible with different regulatory models. Both the sense and antisense sequence motifs could bind with known DNA motifs to form DNA:RNA triplexes that regulate gene expression as above. Alternatively, it could also be the transcription of the intergenic ncRNAs themselves that interferes with the binding of transcription factors to target sites in promoter regions. It has been reported that sequence motifs are widely distributed in the 3′ UTRs of protein-coding genes. They tend to be recognition sites of RNA-binding proteins or target sites of miRNAs, which play important function in mRNA stability or degradation [54]. The existence of sequence motifs in intergenic ncRNAs indicates that a similar regulatory system may also involve non-coding RNAs.

### Expression correlation and functional significance

The poor expression correlation between intergenic ncRNAs and their neighbour genes does not mean that they lack functional significance. There are three arguments that support this view. First the observed dynamic range of MPSS tag abundance for intergenic ncRNAs was very similar to that of RefSeq tags. This implies that similar levels or types of regulation exist for intergenic ncRNAs and mRNAs. Second, the bovine MPSS expression profiles we analysed were generated from multiple sources, including different tissues/cell lines, different developmental stages and different sexes [24]. Studies have confirmed that intergenic ncRNAs tend to be expressed in tissue-specific or development-specific ways [55], [56]. Intergenic ncRNAs in different tissues or developmental stages may be either repressed or activated. This will make the expression correlation fuzzy and unpredictable when these stages are pooled for analysis. Third, intergenic ncRNAs might represent a wide spectrum of functional non-coding RNAs. Different classes of ncRNAs use different mechanisms to regulate gene expression. Some intergenic ncRNAs that are *cis*-regulators might have strong correlations with their neighbour genes. While intergenic ncRNAs functioning in *trans* might show poor correlation with their neighbour genes. The MIC scores for each intergenic ncRNA with all RefSeqs confirmed that many intergenic ncRNAs showed strong correlations with a number of non-neighbour protein-coding genes, which indicated that intergenic ncRNAs might have multiple targets and be involved in multiple gene-regulation networks. In human, mouse and zebrafish, studies based on RNA-seq have also shown that there is no strong expression correlation between intergenic ncRNAs and neighbour genes at the global level [55], [56].

In conclusion, we have demonstrated that EST data sets can be useful for identifying ncRNAs or ncRNA precursors. Genomic distribution and conservation analysis of ncRNAs suggested that these transcripts were not of trivial origin and most originated from genomic regions exhibiting signatures of negative selection or conservation. Our results support the view that most ncRNAs are functional in the context of the regulon hypothesis [57] and that further studies should be aimed at validating this experimentally. Finally we speculate that some of the gene proximate ncRNAs we have identified may act as *cis*-regulatory gene expression elements of regulatory genes through some as yet unknown mechanism(s), but that most of them may be *trans*-acting.

## Supporting Information

Materials S1 **Supporting results and methods.**
(DOCX)

Figure S1 **Classification of** ***cis*** **-NATs identified by pipeline.** The top line denotes three sequentially distributed gene models, in which arrows represent the direction of transcription.
(TIF)

Figure S2 **Most ncRNAs are still conserved after removed UTR-related RNAs.** “URTs” represent “UTR-related RNAs”, which include 4,584 intergenic ncRNAs.
(TIF)

Figure S3 **Three sequence motifs from 5′ intergenic ncRNAs with strong similarity against known DNA motifs.** For each comparison, the upper motif is the known DNA motif, and the lower one is the sequence motif from intergenic ncRNA.
(TIF)

Figure S4 **Four sequence motifs from 3′ intergenic ncRNAs with strong similarity against known DNA motifs.** For each comparison, the upper motif is the known DNA motif, and the lower one is the sequence motif from intergenic ncRNA.
(TIF)

Figure S5 **The sequence motifs identified from intergenic ncRNAs tend to have equal numbers of sense and antisense target sites.** The target site means the sequence region of the motif in its host intergenic ncRNA.
(TIF)

Figure S6 **Expression profiles of “motif and regulatory” intergenic ncRNAs and corresponding neighbour genes across different libraries.** The “motif and regulatory” represents intergenic ncRNA with motif(s) and regulatory neighbour gene. The dots linked with coloured line represent the expresion of one intergenic ncRNA and its neighbour gene across different libraries. A represents 5′ end UTR-related RNAs. B represent 5′ end intergenic ncRNAs with UTR-related RNAs removed. C represent 3′ end UTR-related RNAs, and D represent intergenic ncRNAs with UTR-related RNAs removed.
(TIF)

Figure S7 **Sequence alignment of bovine “** ***ZNFX1-AS1*** **-like” ncRNA and four different human “** ***ZNFX1-AS1*** **” transcript variants.**
(PDF)

Figure S8 **Genomic overview of bovine “** ***ZNFX1-AS1*** **-like” intergenic ncRNA.** The genomic location of bovine “*ZNFX1-AS1*-like” intergenic ncRNA and corresponding protein-coding gene “*ZNFX1*” is shown in A. The zoomed in view of “*ZNFX1-AS1*-like” ncRNA is shown in B.
(TIF)

Table S1 **Library information of all bovine ESTs.** This table contains a detailed description of bovine EST libraries downloaded from NCBI.
(XLSX)

Table S2 **Summary of the programs used in the pipeline.**
(DOCX)

Table S3 **Functional over-representation of the neighbour genes of intergenic ncRNAs.** This table contains the over-represented GO terms for the 5′ end and 3′ end neighbour genes as well as 10 control gene sets for each end.
(XLSX)

Table S4 **Genome coordinates of predicted bovine ncRNAs.** This excel table contains two sheets: The first one is the genomic coordinate file with PSL format and based on genome assembly bosTau4; the second one is the annotation for the intergenic ncRNAs.
(XLSX)

Table S5 **Summary of annotated known ncRNAs.**
(DOCX)

Table S6 **Known ncRNAs identified by Rfam and NONCODE2.0.** This excel table contains ncRNA annotation based on Rfam and NONCODE2.0.
(XLSB)

Table S7 **Summary of identified** ***cis*** **-NATs.** This excel table contains all the known *cis*-NATs that were identified from bovine ESTs.
(XLSX)

Table S8 **Summary of the motifs identified from intergenic ncRNAs.** This excel table contains 4 sheets: motifs identified from all 5′ end intergenic ncRNAs with neighbour genes in less than 5 kb distance; motifs identified from all 3′ end intergenic ncRNAs with neighbour genes in less than 5 kb distance; motifs identified from 5′ end intergenic ncRNAs with UTR-related RNAs removed; motifs identified from 3′ end intergenic ncRNAs with UTR-related RNAs removed.
(XLSX)

Table S9 **Summary of significantly correlated genes with 5′ end intergenic ncRNAs and 3′ end intergenic ncRNAs.** This excel table contains results of genome wide MINE correlation analysis for the 5′ end and 3′ end intergenic ncRNAs.
(XLSX)
