# Supplementary material for: Bovine ncRNAs Are Abundant, Primarily Intergenic, Conserved and Associated with Regulatory Genes
Source: PLoS One. 2012 Aug 6;7(8):e42638. doi: 10.1371/journal.pone.0042638 (PMC3412814; doi:10.1371/journal.pone.0042638)
Supplement: Materials S1 — Supporting results and methods. (DOCX) [file pone.0042638.s001.docx]

**Few well-characterized ncRNAs were identified**

5 ncRNAs were identified as the putative primary transcripts of mir-133b, bta-mir-1-2, bta-mir-2362, bta-mir-2287 and bta-mir-2369 using BLAST against both pre-miRNAs and mature-miRNA databases. Based on Rfam, another ncRNA, CL37604Contig1, was annotated as encoding mir-1306, which had not been identified from bovine in miRBase (Table S6). These primary miRNA transcripts (except CL37604) were also confirmed by manually checking whether they shared the same genomic loci with their corresponding miRNAs in miRBase. We also observed that 5 of these primary miRNA transcripts were unspliced.

In addition to miRNAs, a further 34 putative ncRNAs were highly similar to 31 different RNA models from Rfam (Table S6). 28 of these 34 ncRNAs were snoRNA transcripts, and interestingly, 8 of them contained more than one snoRNA. The most striking example of this was ncRNA “CL17302Contig1”, with four different snoRNAs (SNORA1, SNORA32, snoZ40, SNORA25) spatially distributed within this ncRNA. The biogenesis of snoRNAs indicates that some genes encoding snoRNAs are non-protein-coding and seem to act exclusively as vectors of snoRNA synthesis. The coordinated accumulation of snoRNAs has also been observed in some housekeeping protein-coding genes [[1](#_ENREF_1)]. Our results confirmed that accumulation of snoRNAs occurs in non-protein-coding RNAs. In addition to snoRNAs, 4 tRNAs, located within 3 un-spliced ncRNAs, were identified. Two of them were from one ncRNA, “CL28499Contig1”, which was mapped to the Bovine Mitochondrial genome. The other two were within two different ncRNAs, both of which were located in the introns of a predicted choline dehydrogenase (CHDH) gene. Furthermore, one ssu_RNA was also detected in the ncRNA dataset, a result that is not surprising because polyadenylation of ribosomal RNA has been observed in human cells[[2](#_ENREF_2)]. Another well-annotated ncRNA, known as a SECIS element (selenocysteine insertion sequence), was also found in this ncRNAs dataset. The remaining Rfam annotated ncRNA is a putative catalytic ribozyme RNA with endonuclease activity, called hammerhead_3.

In addition to known ncRNAs in Rfam, an additional 37 putative known ncRNAs were identified from a similarity search against NONCODE2.0 (Table S6). Of these, 22 had significant sequence similarity with mRNA-like ncRNAs, and 15 were identical with known piRNAs.

***Cis*-NATs identified from ESTs**

One additional class of ncRNAs that we identified were *cis*-NATs (Natural Antisense Transcripts). NATs are a class of RNAs that contain the reverse complements of other transcripts from normally functional genes and can regulate these genes by hybridizing to them and forming double stranded RNA. NATs can originate from the same genomic locus as their reverse complement (called *cis*), or from different genomic loci (called *trans*)[[3](#_ENREF_3)]. NATs have been identified from several different species and are believed to regulate gene expression levels via genomic imprinting, RNA alternative splicing and transcription interference [[3](#_ENREF_3),[4](#_ENREF_4),[5](#_ENREF_5)].

We identified 74 *cis-*NAT*s* distributed on 28 different chromosomes (Table S7). When the genomic organization of these 74 *cis*-NATs with their corresponding sense partners was analysed, 5 different types of structures were observed (Figure S1): 1) “head-head”, in which *cis*-NAT is overlapping with gene at 5’ ends; 2) “tail-tail”, in which *cis*-NAT is overlapping with gene at 3’ ends; 3) “contained”, in which the whole *cis*-NAT lies between the transcription stop and start sites of the corresponding gene; 4) “included”, in which the gene lies between the transcription stop and start of the *cis*-NAT; 5) “multiple”, in which the *cis*-NAT overlaps with at least two genes, at least one of which is transcribed from the opposite strand of the *cis*-NAT. The largest class of *cis*-NATs was “contained” (36 of 74). 20, 9 and 8 *cis*-NATs were classified as “tail-tail”, “head-head” and “multiple” respectively. Only 1 *cis*-NAT was classed as “included”. When we classified these *cis*-NATs based on the overlapping regions of their sense genes, we found that 35 of them only overlapped with intronic regions of genes and 1 overlapped only with exonic region. 2 *cis*-NATs overlapped only with gene UTRs. In addition, 6 *cis*-NATs overlapped with exon, intron as well as UTR regions, 26 *cis*-NATs overlapped with exons and introns and 4 *cis*-NATs overlapped with intron and UTR regions (Table S7).

**Supplemental Methods: Identification of *cis*-NATs**

The transcription orientation of unique transcripts was determined with two methods: First, most ESTs have transcription orientation in the header of fasta format sequences. The transcription orientation of contigs was deduced from the individual assembled ESTs. Second, GMAP mapping of transcripts onto the genome, provided the transcription orientation if the transcript had more than 1 exon.

Only transcripts with the same orientation according to both of the above methods were used to identify *cis*-NATs. Bovine RefSeqs and all transcripts with definite transcription orientation were mapped to the genome as well. A Perl script was created to extract the sense-antisense pairs based on the genomic locations of ncRNAs and RefSeqs. All candidate sense-antisense pairs were manually validated with the UCSC genome browser by checking their genomic loci.

Supplemental References

1. Filipowicz W, Pogacic V (2002) Biogenesis of small nucleolar ribonucleoproteins. Curr Opin Cell Biol 14: 319-327.

2. Slomovic S, Laufer D, Geiger D, Schuster G (2006) Polyadenylation of ribosomal RNA in human cells. Nucleic Acids Res 34: 2966-2975.

3. Lavorgna G, Dahary D, Lehner B, Sorek R, Sanderson CM, et al. (2004) In search of antisense. Trends Biochem Sci 29: 88-94.

4. Li JT, Zhang Y, Kong L, Liu QR, Wei L (2008) Trans-natural antisense transcripts including noncoding RNAs in 10 species: implications for expression regulation. Nucleic Acids Res 36: 4833-4844.

5. Zhang Y, Liu XS, Liu QR, Wei L (2006) Genome-wide in silico identification and analysis of cis natural antisense transcripts (cis-NATs) in ten species. Nucleic Acids Res 34: 3465-3475.

6. Wu TD, Watanabe CK (2005) GMAP: a genomic mapping and alignment program for mRNA and EST sequences. Bioinformatics 21: 1859-1875.

7. Rice P, Longden I, Bleasby A (2000) EMBOSS: the European Molecular Biology Open Software Suite. Trends Genet 16: 276-277.
