# Supplementary figures and images for: Bovine ncRNAs Are Abundant, Primarily Intergenic, Conserved and Associated with Regulatory Genes
Source: PLoS One. 2012 Aug 6;7(8):e42638. doi: 10.1371/journal.pone.0042638 (PMC3412814; doi:10.1371/journal.pone.0042638)

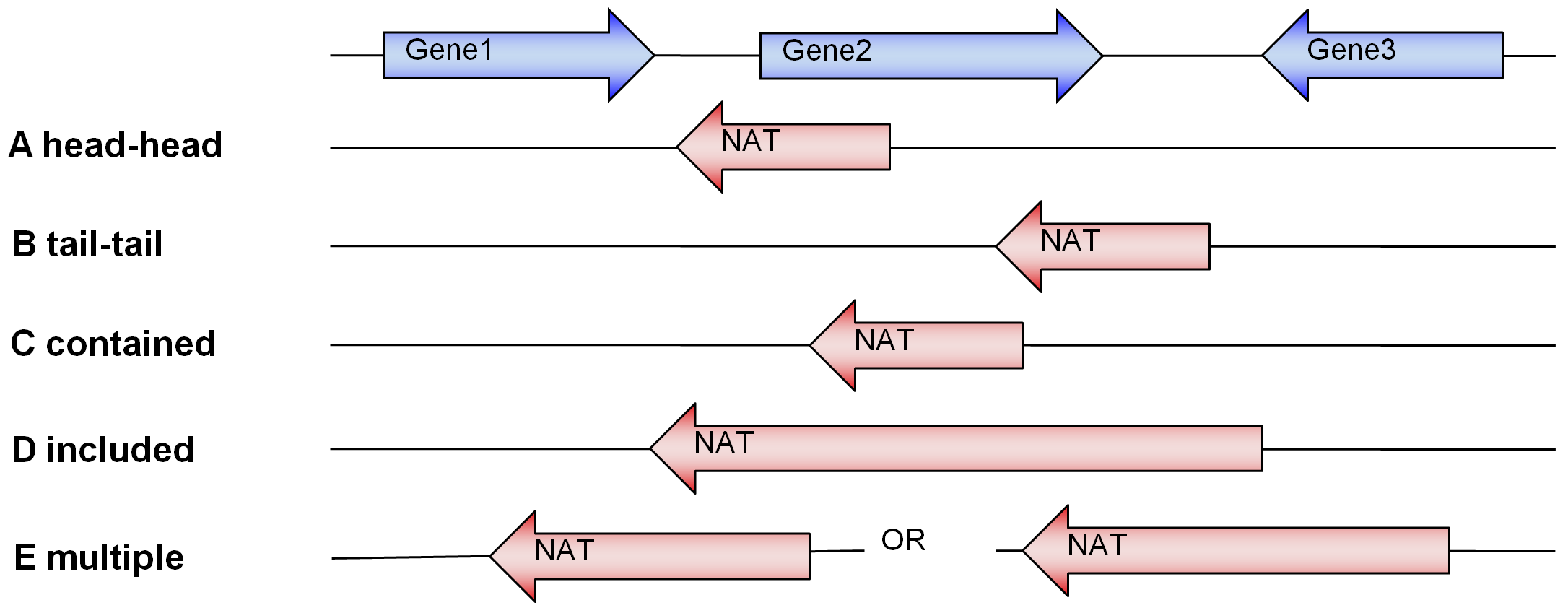

Supplement: Figure S1 — Classification of cis -NATs identified by pipeline. The top line denotes three sequentially distributed gene models, in which arrows represent the direction of transcription. (TIF) [file pone.0042638.s002.tif]

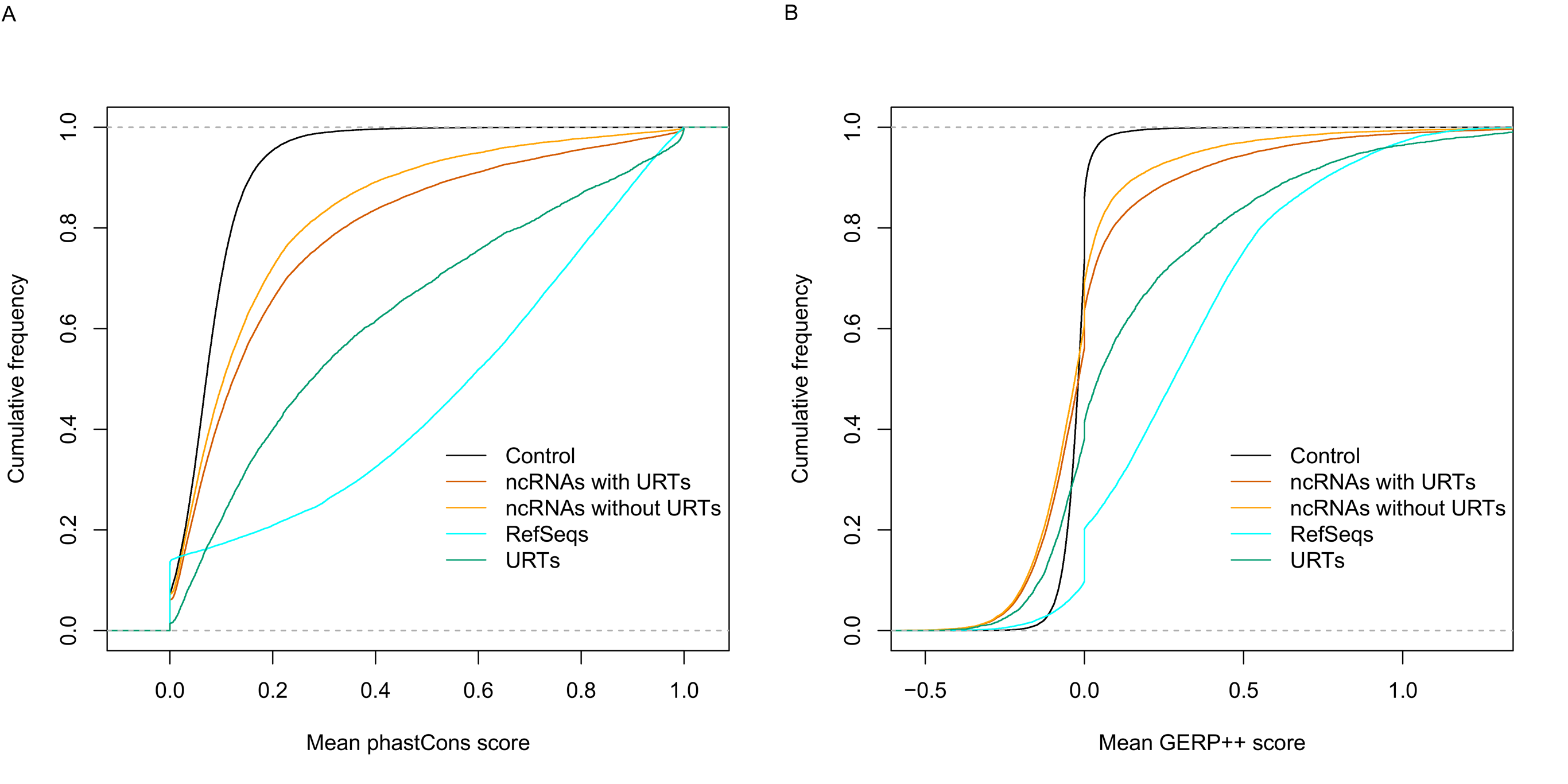

Supplement: Figure S2 — Most ncRNAs are still conserved after removed UTR-related RNAs. “URTs” represent “UTR-related RNAs”, which include 4,584 intergenic ncRNAs. (TIF) [file pone.0042638.s003.tif]

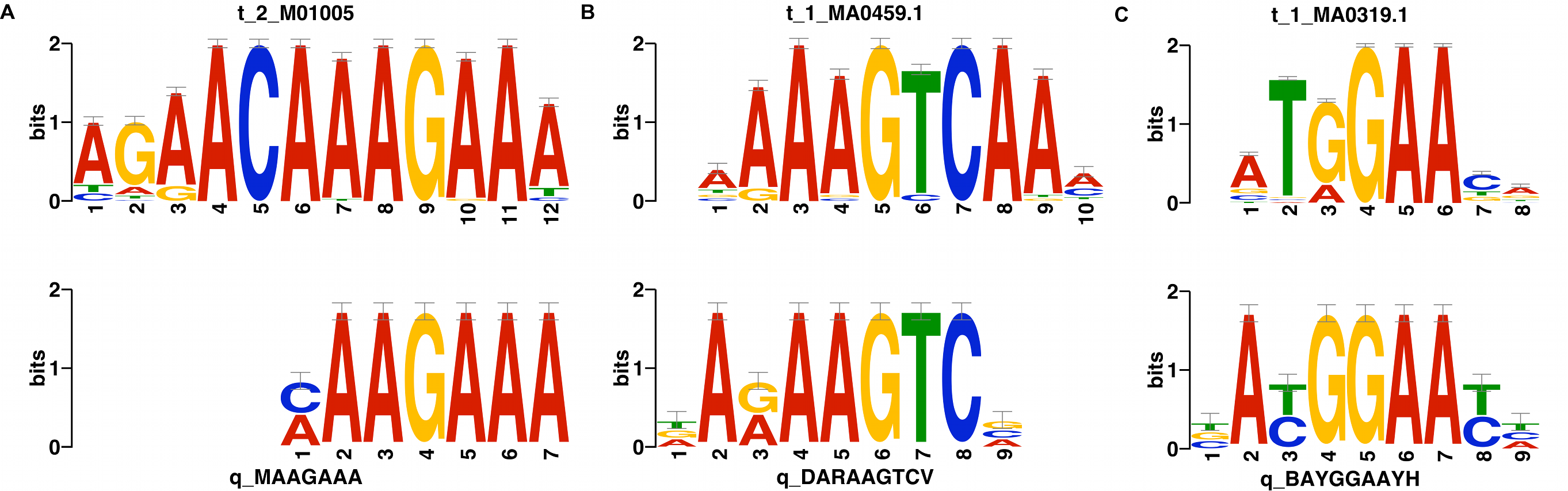

Supplement: Figure S3 — Three sequence motifs from 5′ intergenic ncRNAs with strong similarity against known DNA motifs. For each comparison, the upper motif is the known DNA motif, and the lower one is the sequence motif from intergenic ncRNA. (TIF) [file pone.0042638.s004.tif]

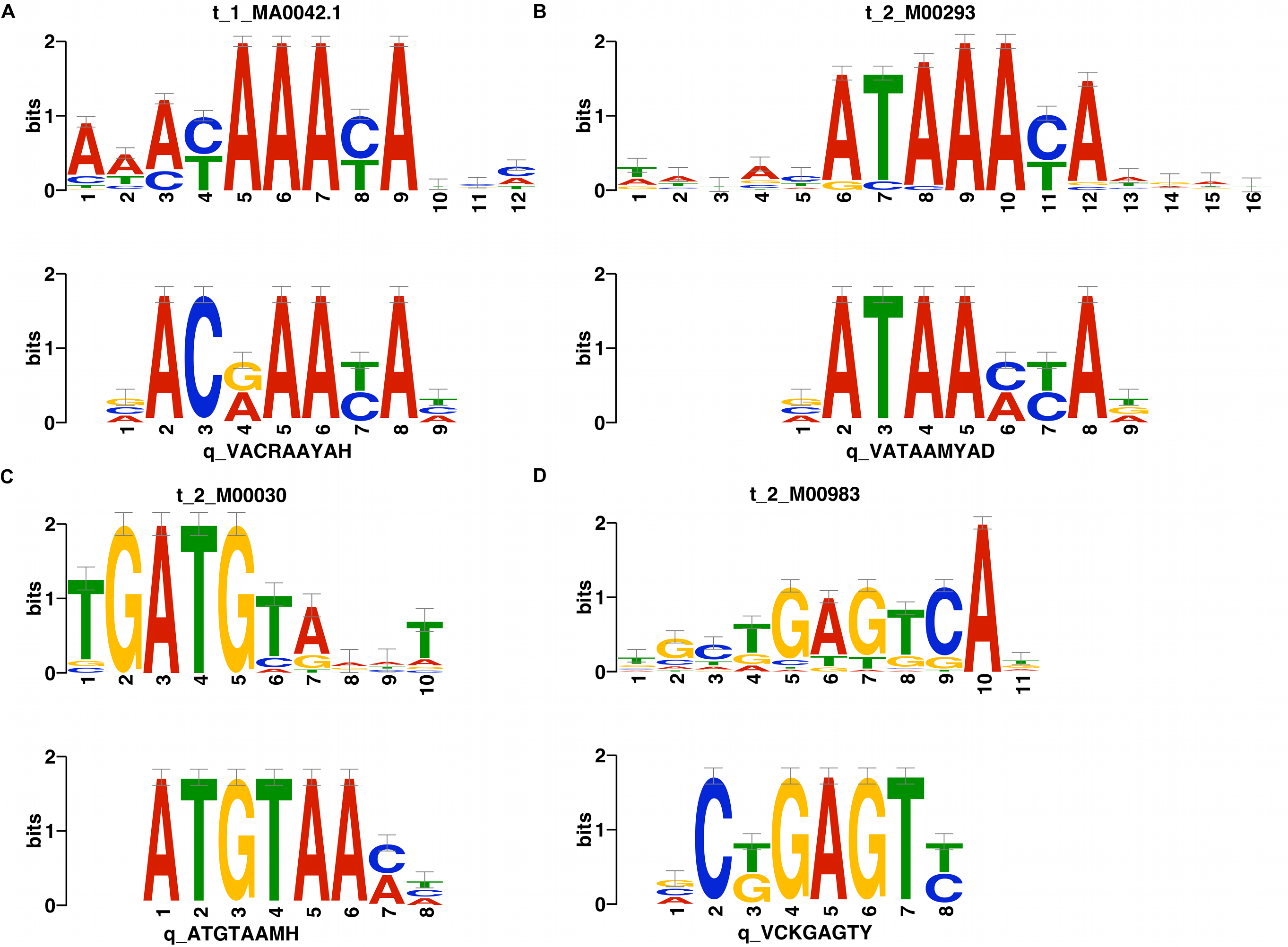

Supplement: Figure S4 — Four sequence motifs from 3′ intergenic ncRNAs with strong similarity against known DNA motifs. For each comparison, the upper motif is the known DNA motif, and the lower one is the sequence motif from intergenic ncRNA. (TIF) [file pone.0042638.s005.tif]

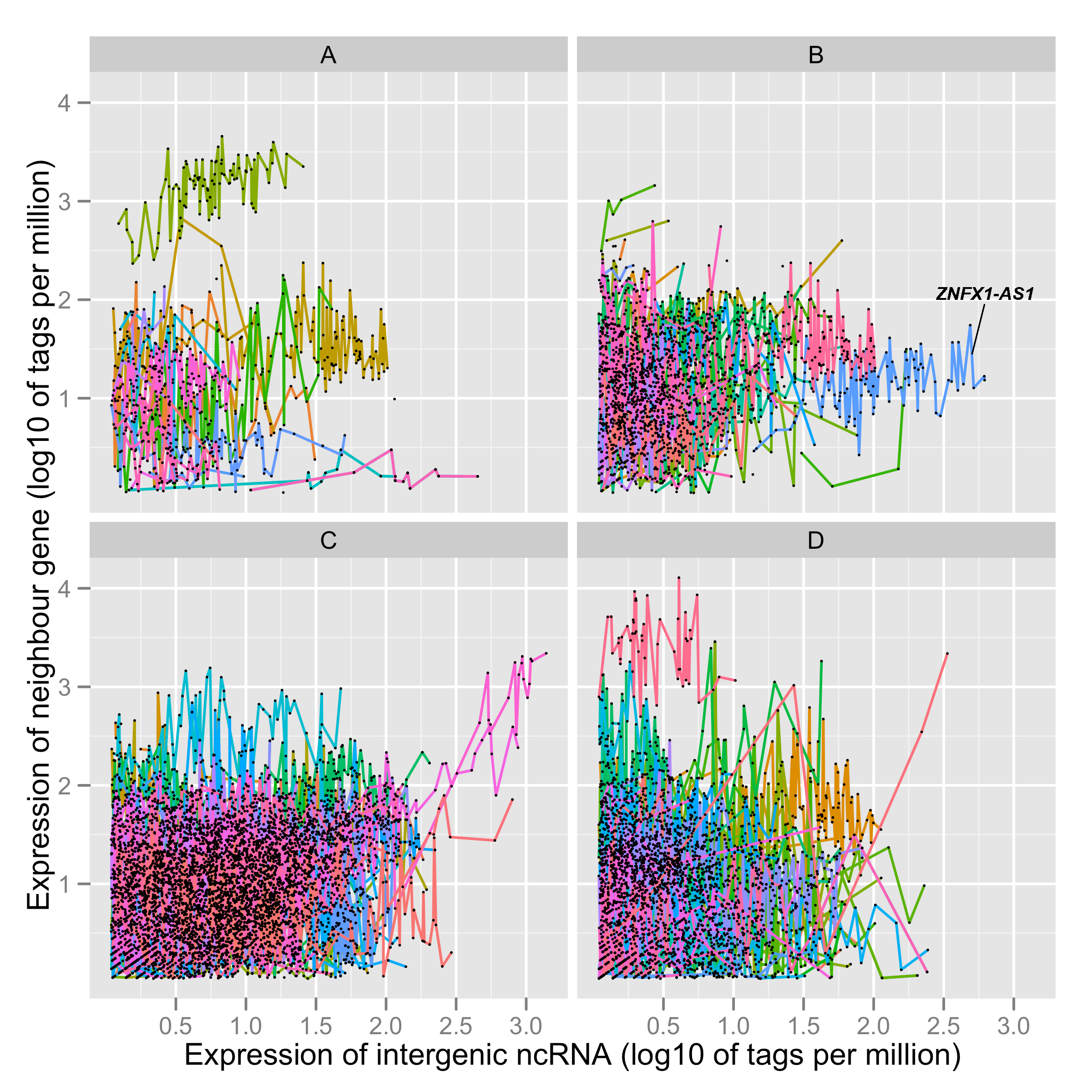

Supplement: Figure S6 — Expression profiles of “motif and regulatory” intergenic ncRNAs and corresponding neighbour genes across different libraries. The “motif and regulatory” represents intergenic ncRNA with motif(s) and regulatory neighbour gene. The dots linked with coloured line represent the expresion of one intergenic ncRNA and its neighbour gene across different libraries. A represents 5′ end UTR-related RNAs. B represent 5′ end intergenic ncRNAs with UTR-related RNAs removed. C represent 3′ end UTR-related RNAs, and D represent intergenic ncRNAs with UTR-related RNAs removed. (TIF) [file pone.0042638.s007.tif]

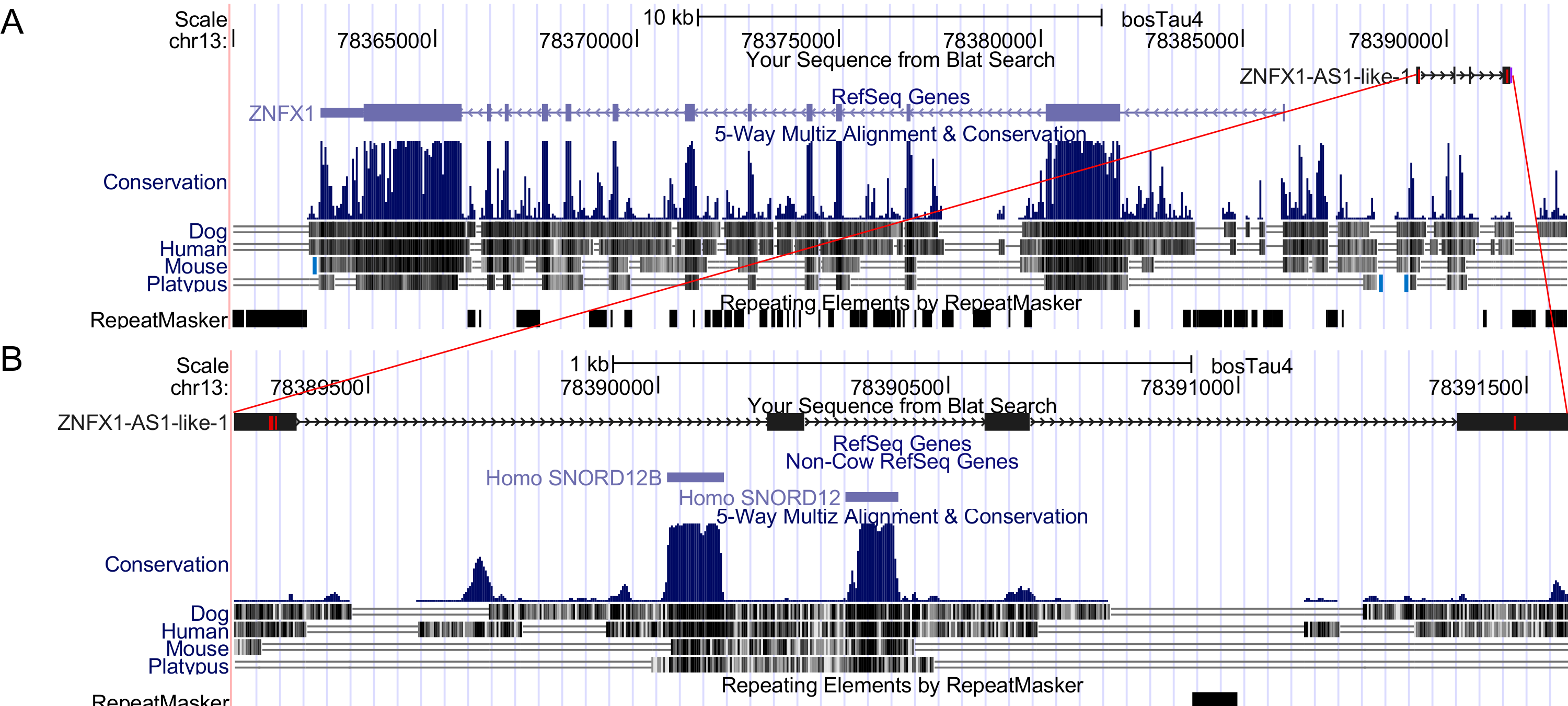

Supplement: Figure S8 — Genomic overview of bovine “ ZNFX1-AS1 -like” intergenic ncRNA. The genomic location of bovine “ZNFX1-AS1-like” intergenic ncRNA and corresponding protein-coding gene “ZNFX1” is shown in A. The zoomed in view of “ZNFX1-AS1-like” ncRNA is shown in B. (TIF) [file pone.0042638.s009.tif]
