# Supplementary material for: Bovine ncRNAs Are Abundant, Primarily Intergenic, Conserved and Associated with Regulatory Genes
Source: PLoS One. 2012 Aug 6;7(8):e42638. doi: 10.1371/journal.pone.0042638 (PMC3412814; doi:10.1371/journal.pone.0042638)

```
Human_ZNFX1AS1-3 CGGGGGCCCAGGGTGGAGAGCACGAGGGCCTGGCCCAGGCACGGCCGGCGCCTCCGCCCTCGAGGAGGGCGTCACCTCAG    80
Human_ZNFX1AS1-4 CGGGGGCCCAGGGTGGAGAGCACGAGGGCCTGGCCCAGGCACGGCCGGCGCCTCCGCCCTCGAGGAGGGCGTCACCTCAG    80
Human_ZNFX1AS1-5 --------------------------------------------------------------------------------
Human_ZNFX1AS1-1 CGGGGGCCCAGGGTGGAGAGCACGAGGGCCTGGCCCAGGCACGGCCGGCGCCTCCGCCCTCGAGGAGGGCGTCACCTCAG    80
Human_ZNFX1AS1-2 --------------------------------------------------------------------------------
ZNFX1-AS1-like-1 --------------------------------------------------------------------------------
                 1.......10........20........30........40........50........60........70........80

Human_ZNFX1AS1-3 CTCCCCCCGGCGGCGGAGCCGGCGGGCTCAGGCGGGCGCGGCTGAGGGGAGCGGACCGCGGGGGGCGGGAGATGACTGCG   160
Human_ZNFX1AS1-4 CTCCCCCCGGCGGCGGAGCCGGCGGGCTCAGGCGGGCGCGGCTGAGGGGAGCGGACCGCGGGGGGCGGGAGATGACTGCG   160
Human_ZNFX1AS1-5 --------------------------------------------------------------------------------
Human_ZNFX1AS1-1 CTCCCCCCGGCGGCGGAGCCGGCGGGCTCAGGCGGGCGCGGCTGAGGGGAGCGGACCGCGGGGGGCGGGAGATGACTGCG   160
Human_ZNFX1AS1-2 --------------------------------------------------------------------------------
ZNFX1-AS1-like-1 --------------------------------------------------------------------------------
                 ........90.......100.......110.......120.......130.......140.......150.......160

Human_ZNFX1AS1-3 CCCAAGGCCTTTGCGGGCCTCAGCCGGCCCCAGAGGAAGGGGAACCCGTCGAGCGGTTTGGTGCGTGTGAAGCGCGACAT   240
Human_ZNFX1AS1-4 CCCAAGGCCTTTGCGGGCCTCAGCCGGCCCCAGAGGAAGGGGAACCCGTCGAGCGGTTTGGTGCGTGTGAAGCGCGACAT   240
Human_ZNFX1AS1-5 --------------------------------------------------------------------------------
Human_ZNFX1AS1-1 CCCAAGGCCTTTGCGGGCCTCAGCCGGCCCCAGAGGAAGGGGAACCCGTCGAGCGGTTTGGTGCGTGTGAAGCGCGACAT   240
Human_ZNFX1AS1-2 --------------------------------------------------------------------------------
ZNFX1-AS1-like-1 --------------------------------------------------------------------------------
                 .......170.......180.......190.......200.......210.......220.......230.......240

Human_ZNFX1AS1-3 GGCGAGGAAGCGGACAAGCCCGGGTGGCCCGGCGTGTAGAGGGAAGGGGGCGGGGCTAGACGCGGCCTGGACAACTACTA   320
Human_ZNFX1AS1-4 GGCGAGGAAGCGGACAAGCCCGGGTGGCCCGGCGTGTAGAGGGAAGGGGGCGGGGCTAGACGCGGCCTGGACAACTACTA   320
Human_ZNFX1AS1-5 --------------------------------------------------------------------------------
Human_ZNFX1AS1-1 GGCGAGGAAGCGGACAAGCCCGGGTGGCCCGGCGTGTAGAGGGAAGGGGGCGGGGCTAGACGCGGCCTGGACAACTACTA   320
Human_ZNFX1AS1-2 --------------------------------------------------------------------------------
ZNFX1-AS1-like-1 --------------------------------------------------------------------------------
                 .......250.......260.......270.......280.......290.......300.......310.......320

Human_ZNFX1AS1-3 GAGCGCCTCGGGCTGTGCTGCTCGAGACTACATTTCCCAGAGCGACGCGCGCGGAGCGGGCGGGAAAGAGAGCGTTTCGG   400
Human_ZNFX1AS1-4 GAGCGCCTCGGGCTGTGCTGCTCGAGACTACATTTCCCAGAGCGACGCGCGCGGAGCGGGCGGGAAAGAGAGCGTTTCGG   400
Human_ZNFX1AS1-5 --------------------------------------------------------------------------------
Human_ZNFX1AS1-1 GAGCGCCTCGGGCTGTGCTGCTCGAGACTACATTTCCCAGAGCGACGCGCGCGGAGCGGGCGGGAAAGAGAGCGTTTCGG   400
Human_ZNFX1AS1-2 CGGGGGCCCAGGGTGGAGAGCACGAGGGCCTGGCCCAGGCACGGCCGGCGCCTCCGCCCTCGAGGAGGGCGTCACCTCAG    80
ZNFX1-AS1-like-1 --------------------------------------------------------------------------------
                 .......330.......340.......350.......360.......370.......380.......390.......400

Human_ZNFX1AS1-3 GTCCAGTGCGCAGGTGCGAAAGCCATCTTTGGTTATATAAGGGAGGTTCAGGAAGCCATTCGTTCTTTCGCGTCTGCGGT   480
Human_ZNFX1AS1-4 GTCCAGTGCGCAGGTGCGAAAGCCATCTTTGGTTATATAAGGGAGGTTCAGGAAGCCATTCGTTCTTTCGCGTCTGCGGT   480
Human_ZNFX1AS1-5 ----------------------------------------------------------------CTTTCGCGTCTGCGGT    16
Human_ZNFX1AS1-1 GTCCAGTGCGCAGGTGCGAAAGCCATCTTTGGTTATATAAGGGAGGTTCAGGAAGCCATTCGTTCTTTCGCGTCTGCGGT   480
Human_ZNFX1AS1-2 CTCCCCCCGGCGGCGGAGCCGGCGGGCTCAGGC-----GGGCGCGGCTGAGGGGAGCG---GACCGCGGGGGGCGGGAGA   152
ZNFX1-AS1-like-1 --------------------------------------------------------------------------------
                 .......410.......420.......430.......440.......450.......460.......470.......480

Human_ZNFX1AS1-3 GCCCGGAGTGTGGTACTTCTCCTAGTTGCAGTCAGGCTTCATACGCTATTGTCCTGCCCGT-------------------   541
Human_ZNFX1AS1-4 GCCCGGAGTGTGGTACTTCTCCTAGTTGCAGTCAGGCTTCATACGCTATTGTCCTGCCCGT-------------------   541
Human_ZNFX1AS1-5 GCCCGGAGTGTGGTACTTCTCCTAGTTGCAGTCAGGCTTCATACGCTATTGTCCTGCCCGTAAGTTCCCGTTTTGTGTGT    96
Human_ZNFX1AS1-1 GCCCGGAGTGTGGTACTTCTCCTAGTTGCAGTCAGGCTTCATACGCTATTGTCCTGCCCGT-------------------   541
Human_ZNFX1AS1-2 TGACTGCGCCCAAGGCCTTTGCGGGCCTCAGCC-GGCCCCAGAGGAAGGGGAACCCGTCGAGCG------------GTTT   219
ZNFX1-AS1-like-1 --------------------------------------------------------------------------------
                 .......490.......500.......510.......520.......530.......540.......550.......560
```

```
                    * * *    ***  * ********* ** ****  ***** *** *  ****  *** **     **** **  ***
Human_ZNFX1AS1-3 ---TAGAGCAGCCAGCGGGTACAGAATGGATTTTGGAAGAGGGAGTCACCACTGGACCTCCAAGGAAGCCACGTGCAGAC  618
Human_ZNFX1AS1-4 ---TAGAGCAGCCAGCGGGTACAGAATGGATTTTGGAAGAGGGAGTCACCACTGGACCTCCAAGGAAGCCACGTGCAGAC  618
Human_ZNFX1AS1-5 GGTTAGAGCAGCCAGCGGGTACAGAATGGATTTTGGAAGAGGGAGTCACCACTGGACCTCCAAGGAAGCCACGTGCAGAC  176
Human_ZNFX1AS1-1 ---TAGAGCAGCCAGCGGGTACAGAATGGATTTTGGAAGAGGGAGTCACCACTGGACCTCCAAGGAAGCCACGTGCAGAC  618
Human_ZNFX1AS1-2 GGTTAGAGCAGCCAGCGGGTACAGAATGGATTTTGGAAGAGGGAGTCACCACTGGACCTCCAAGGAAGCCACGTGCAGAC  299
ZNFX1-AS1-like-1 -ATTTGCGTGATCAGACGCTACAGAATGAATCTTGGCGGAGGG-GTCCCTGCTGGGGCTCAAATCTCTCCACATGTCGAC   78
                 .......570.......580.......590.......600.......610.......620.......630.......640
```

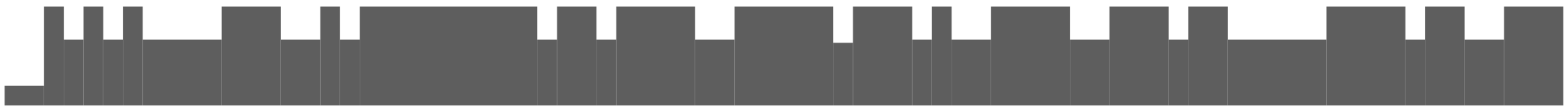

```
                 *   *********    *******     **
Human_ZNFX1AS1-3 ATCTACAACCTTCGATCTCCTGAC-----GA-------------------------------------------------  644
Human_ZNFX1AS1-4 ATCTACAACCTTCGATCTCCTGAC-----GAGTTTATTGTTGGCCAAAACCAGGCTTTGATTGAACCAGGATGAATGCGG  693
Human_ZNFX1AS1-5 ATCTACAACCTTCGATCTCCTGAC-----GAGTTTATTGTTGGCCAAAACCAGGCTTTGATTGAACCAGGATGAATGCGG  251
Human_ZNFX1AS1-1 ATCTACAACCTTCGATCTCCTGAC-----GAGTTTATTGTTGGCCAAAACCAGGCTTTGATTGAACCAGGATGAATGCGG  693
Human_ZNFX1AS1-2 ATCTACAACCTTCGATCTCCTGAC-----GAGTTTATTGTTGGCCAAAACCAGGCTTTGATTGAACCAGGATGAATGCGG  374
ZNFX1-AS1-like-1 AGGCACAACCTTCAGCTTCCTGACCTCTGGAGCTCACTGTTGGCAGCAAACAGGCATTGAATGGACCATGATGGATCCAG  158
                 .......650.......660.......670.......680.......690.......700.......710.......720
```

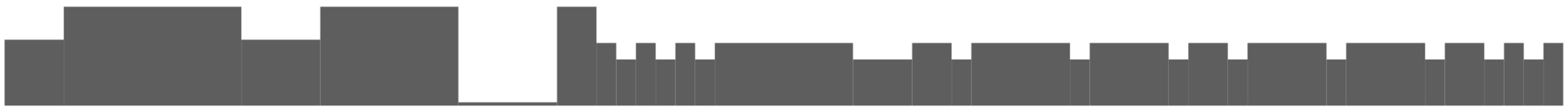

```
Human_ZNFX1AS1-3 --------------------------------------------------------------------------------  644
Human_ZNFX1AS1-4 GTGTTGGAAGTAGAATATATATATACATATAAAATTG-------------------------------------------  730
Human_ZNFX1AS1-5 GTGTTGGAAGTAGAATATATATATACATATAAAATTG-------------------------------------------  288
Human_ZNFX1AS1-1 GTGTTGGAAGTAGAATATATATATACATATAAAATTGGTTGGGAGCCACGTGTACCAGTGTGTGTTGATCTTGGCTTGAT  773
Human_ZNFX1AS1-2 GTGTTGGAAGTAGAATATATATATACATATAAAATTGGTTGGGAGCCACGTGTACCAGTGTGTGTTGATCTTGGCTTGAT  454
ZNFX1-AS1-like-1 GATTGGGAGAGAATTTGACCAGAGCCCTGTGTACATCAGGGCCTACATAGGTTGATGGCAGCCTGATTGCAGTTTGGTTC  238
                 .......730.......740.......750.......760.......770.......780.......790.......800
```

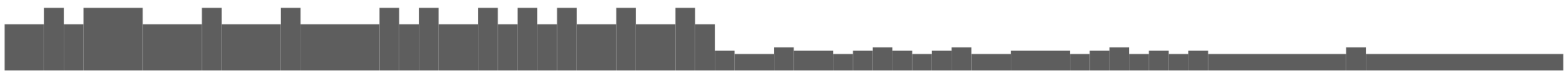

```
                                     *****  **   *  ***  ***   ***** ** * *** * ***    **       *
Human_ZNFX1AS1-3 -------------------AAACTGGCGATGGAATATGAGAGGAGCCCTCTGGAAAGAAAAGGACAGACCCTGTGCTTTC  705
Human_ZNFX1AS1-4 -------------------AAACTGGCGATGGAATATGAGAGGAGCCCTCTGGAAAGAAAAGGACAGACCCTGTGCTTTC  791
Human_ZNFX1AS1-5 -------------------AAACTGGCGATGGAATATGAGAGGAGCCCTCTGGAAAGAAAAGGACAGACCCTGTGCTTTC  349
Human_ZNFX1AS1-1 TCAGTCTGCCTTGTAACAGAAACTGGCGATGGAATATGAGAGGAGCCCTCTGGAAAGAAAAGGACAGACCCTGTGCTTTC  853
Human_ZNFX1AS1-2 TCAGTCTGCCTTGTAACAGAAACTGGCGATGGAATATGAGAGGAGCCCTCTGGAAAGAAAAGGACAGACCCTGTGCTTTC  534
ZNFX1-AS1-like-1 AGTTCTGTAACAGGAGTTTCAACTGTGGAGTCAGAATGCAAGG---CCTCTTGAGAAAAATGTACATTTACTTCTGAAGC  315
                 .......810.......820.......830.......840.......850.......860.......870.......880
```

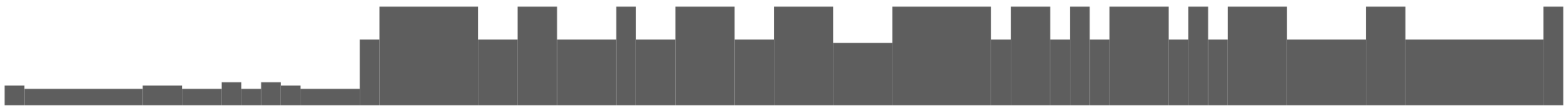

```
                 *****   ***** * *** *      ***    **  * ***** **        *** * *****  **** *   **
Human_ZNFX1AS1-3 ATGAAA-GTGAAGATCTGGCTGAACCAGTTCCACAAGGTTACTGTATACATAGCCTGAGTTTAAAAGGCTGTGCCCACTT  784
Human_ZNFX1AS1-4 ATGAAA-GTGAAGATCTGGCTGAACCAGTTCCACAAGGTTACTGTATACATAGCCTGAGTTTAAAAGGCTGTGCCCACTT  870
Human_ZNFX1AS1-5 ATGAAA-GTGAAGATCTGGCTGAACCAGTTCCACAAGGTTACTGTATACATAGCCTGAGTTTAAAAGGCTGTGCCCACTT  428
Human_ZNFX1AS1-1 ATGAAA-GTGAAGATCTGGCTGAACCAGTTCCACAAGGTTACTGTATACATAGCCTGAGTTTAAAAGGCTGTGCCCACTT  932
Human_ZNFX1AS1-2 ATGAAA-GTGAAGATCTGGCTGAACCAGTTCCACAAGGTTACTGTATACATAGCCTGAGTTTAAAAGGCTGTGCCCACTT  613
ZNFX1-AS1-like-1 ATGAAGCATGAAGGTTTGGGTT-----GTTGTGTAACCTAACTGTGTA--------GAGGTAAAAAGCTTGTGGCTGTTT  382
                 .......890.......900.......910.......920.......930.......940.......950.......960
```

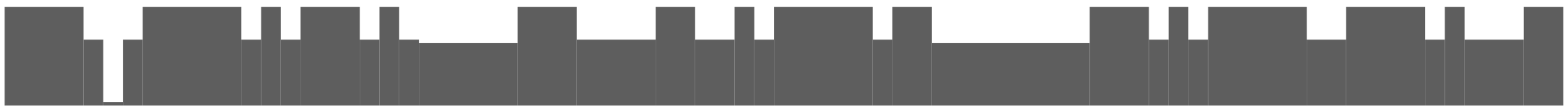

```
                            ** **  ****** *   *       ***  **  * *** *********** ******* ********
Human_ZNFX1AS1-3 CAAGAATGTCATTGTTAGACTTTGAAATTTCTAACTGCCTACCTGCATAAAGAAAATAAAATCTTTTAAATCAAAAAAAA  864
Human_ZNFX1AS1-4 CAAGAATGTCATTGTTAGACTTTGAAATTTCTAACTGCCTACCTGCATAAAGAAAATAAAATCTTTTAAATCAAAAAAAA  950
Human_ZNFX1AS1-5 CAAGAATGTCATTGTTAGACTTTGAAATTTCTAACTGCCTACCTGCATAAAGAAAATAAAATCTTTTAAATCAAAAAAAA  508
Human_ZNFX1AS1-1 CAAGAATGTCATTGTTAGACTTTGAAATTTCTAACTGCCTACCTGCATAAAGAAAATAAAATCTTTTAAATCAAAAAAAA 1012
Human_ZNFX1AS1-2 CAAGAATGTCATTGTTAGACTTTGAAATTTCTAACTGCCTACCTGCATAAAGAAAATAAAATCTTTTAAATCAAAAAAAA  693
ZNFX1-AS1-like-1 TGG--------TTATTTTACTTTGTAGCCTT------CCTGTCTAGAGAAATAAAATAAAATCCTTTAAATGAAAAAAAA  448
                 .......970.......980.......990......1000......1010......1020......1030......1040
```

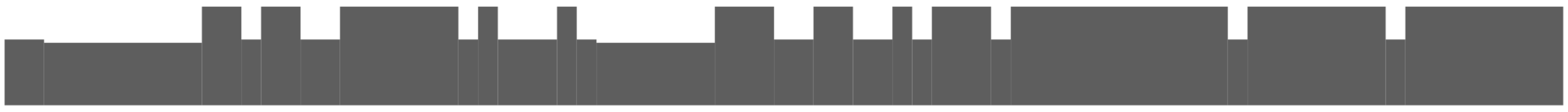

```
                 **
Human_ZNFX1AS1-3 AAAAAAAA-------   872
Human_ZNFX1AS1-4 AAAAAAAAA------   959
Human_ZNFX1AS1-5 AAAAAAAA-------   516
Human_ZNFX1AS1-1 AAAAAAAA-------  1020
Human_ZNFX1AS1-2 AA-------------   695
ZNFX1-AS1-like-1 AAAAAAAAATTCGGA   463
                 ......1050.....
```

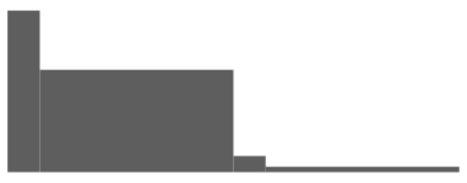

Supplement: Figure S7 — Sequence alignment of bovine “ ZNFX1-AS1 -like” ncRNA and four different human “ ZNFX1-AS1 ” transcript variants. (PDF) [file pone.0042638.s008.pdf]
