# Supplementary material for: Bovine ncRNAs Are Abundant, Primarily Intergenic, Conserved and Associated with Regulatory Genes
Source: PLoS One. 2012 Aug 6;7(8):e42638. doi: 10.1371/journal.pone.0042638 (PMC3412814; doi:10.1371/journal.pone.0042638)
Supplement: Table S2 — Summary of the programs used in the pipeline. (DOCX) [file pone.0042638.s011.docx]

**Table S2** Summary of the programs used in the pipeline

| **Name** | **Function in pipeline** | **Website/Reference** |
| --- | --- | --- |
| SEQCLEAN | EST quality control | http://compbio.dfci.harvard.edu/tgi/software/ |
| RepeatMasker | Screen repeats | http://www.repeatmasker.org/ |
| WU_BLAST* | Similarity search | http://blast.wustl.edu/ |
| TGICL | EST clustering and assembly | http://compbio.dfci.harvard.edu/tgi/software/ |
| GMAP | Genome mapping | [[6](#_ENREF_6)]http://research-pub.gene.com/gmap/ |
| NCBI_BLAST | Similarity search | ftp://ftp.ncbi.nih.gov/blast/ |
| EMBOSS (getorf) | ORF prediction | [[7](#_ENREF_7)]http://emboss.sourceforge.net/ |

* WU_BLAST has been renamed to AB_BLAST, which is not freely accessible. We used version 2 of WU_BLAST in the pipeline.
