# Supplementary material for: Bovine ncRNAs Are Abundant, Primarily Intergenic, Conserved and Associated with Regulatory Genes
Source: PLoS One. 2012 Aug 6;7(8):e42638. doi: 10.1371/journal.pone.0042638 (PMC3412814; doi:10.1371/journal.pone.0042638)
Supplement: Table S5 — Summary of annotated known ncRNAs. (DOCX) [file pone.0042638.s014.docx]

**Table S5** Summary of annotated known ncRNAs

| **Class** | **Number** | **Databases/Programs** |
| --- | --- | --- |
| miRNA | 6 | miRBase, Rfam |
| snoRNA | 28 | Rfam |
| tRNA | 3 | tRNAscan_SE, Rfam |
| rRNA | 1 | ARAGORN, Rfam |
| mRNA-like ncRNA | 22 | NONCODE2.0 |
| piRNA | 15 | NONCODE2.0 |
| other | 2 | Rfam |
